# Comparison of methods for the detection of outliers and associated biomarkers in mislabeled omics data

**DOI:** 10.1186/s12859-020-03653-9

**Published:** 2020-08-14

**Authors:** Hongwei Sun, Yuehua Cui, Hui Wang, Haixia Liu, Tong Wang

**Affiliations:** 1 Department of Health Statistics, School of Public Health, Shanxi Medical University, Taiyuan City, 030001 Shanxi China; 2 Department of Health Statistics, School of Public Health and Management, Binzhou Medical University, City, Yantai, 264003 Shandong China; 3 Department of Statistics and Probability, Michigan State University, East Lansing, MI 48824 USA

**Keywords:** Rlogreg, enetLTS, Ensemble, Mislabeled, Robust, Feature selection

## Abstract

**Background:**

Previous studies have reported that labeling errors are not uncommon in omics data. Potential outliers may severely undermine the correct classification of patients and the identification of reliable biomarkers for a particular disease. Three methods have been proposed to address the problem: sparse label-noise-robust logistic regression (Rlogreg), robust elastic net based on the least trimmed square (enetLTS), and Ensemble. Ensemble is an ensembled classification based on distinct feature selection and modeling strategies. The accuracy of biomarker selection and outlier detection of these methods needs to be evaluated and compared so that the appropriate method can be chosen.

**Results:**

The accuracy of variable selection, outlier identification, and prediction of three methods (Ensemble, enetLTS, Rlogreg) were compared for simulated and an RNA-seq dataset. On simulated datasets, Ensemble had the highest variable selection accuracy, as measured by a comprehensive index, and lowest false discovery rate among the three methods. When the sample size was large and the proportion of outliers was ≤5%, the positive selection rate of Ensemble was similar to that of enetLTS. However, when the proportion of outliers was 10% or 15%, Ensemble missed some variables that affected the response variables.

Overall, enetLTS had the best outlier detection accuracy with false positive rates *<* 0.05 and high sensitivity, and enetLTS still performed well when the proportion of outliers was relatively large. With 1% or 2% outliers, Ensemble showed high outlier detection accuracy, but with higher proportions of outliers Ensemble missed many mislabeled samples. Rlogreg and Ensemble were less accurate in identifying outliers than enetLTS. The prediction accuracy of enetLTS was better than that of Rlogreg. Running Ensemble on a subset of data after removing the outliers identified by enetLTS improved the variable selection accuracy of Ensemble.

**Conclusions:**

When the proportion of outliers is ≤5%, Ensemble can be used for variable selection. When the proportion of outliers is > 5%, Ensemble can be used for variable selection on a subset after removing outliers identified by enetLTS. For outlier identification, enetLTS is the recommended method. In practice, the proportion of outliers can be estimated according to the inaccuracy of the diagnostic methods used.

## Background

With the development of high-throughput technologies, an increasing amount of high-dimensional genomic data is being generated. The problem of determining diagnostic, prognostic, and therapeutic markers for disease and clinical outcomes has been addressed by many methods. These methods can manage a high-dimensional and low sample size setting, and include regularized methods, such asthe elastic net (EN) [1], least absolute shrinkage and selection operator (LASSO) [2]; and feature extraction methods, such as partial least squares (PLS) regression [3].

The performance of these methods depends on the accurate labeling of data. Mislabeled samples would deteriorate the accuracy of these procedures seriously [4]. However, there is no guarantee that the class labels are all correct. Researchers have reported that 10–15% of samples are mislabeled in a microarray [5, 6]. For example, an RNA-seq dataset for triple negative breast cancer (TNBC) [7] contains 28 samples with discordant labels obtained from different tests (immunohistochemical (IHC) method or fluorescence in situ hybridization (FISH)), which are potential outliers. Genomic data typically incorporates mislabeled samples that arrive from many sources, such as a missed diagnosis or misdiagnosis, and samples mislabeled in experiments [8]. Moreover, technical problems in microarray experiments or heterogeneity problems, such as samples obtained from different subpopulations, result in outliers [8].

It is crucial to detect outliers. Associated marker selection and the prediction of patient outcome would not be influenced by label noise. Additionally, wrongly diagnosed patients that are detected and treated may receive appropriate treatment instead of ineffective treatment, or even harmful treatment. Moreover, if detected outliers are not mislabeled after being checked, they may be unusual clinical cases that may reveal hidden information on the covariate and probably be worth studying further [9].

Many feature selection methods exist for high-dimensional omics data [1, 2]. However, there are very few feature selection methods that consider the problem of mislabeled samples.

Bootkrajang et al. [4] proposed sparse label-noise-robust logistic regression (Rlogreg) to detect mislabeled arrays with a sparse logistic regression classifier. Rlogreg is a robust extension of the Bayesian logistic regression classifier (Blogreg) proposed by Shevade and Keerthi [10]. In Rlogreg, a label-flipping probability that accounts for possible mislabeling is defined as part of the classifier. Bayesian regularization is used to set the regularization parameter instead of cross-validation to save computational time and eliminate the effects of label noise when setting the regularization parameter.

Kurnaz et al. [11] proposed the robust elastic net based on the least trimmed square (enetLTS), which is a robust method for linear and logistic regression based on the EN penalty. The least trimmed square (LTS) is used to obtain robust results, and the EN penalty allows for variable selection in high-dimensional sparse settings. The trimming procedure is highly robust, but also leads to a loss in efficiency, and therefore, a reweighted step is considered in enetLTS.

To detect outliers in high-dimensional datasets including mislabeled samples, Lopes et al. [7] proposed Ensemble, which is an ensemble classification setting based on distinct procedures, including logistic regression with elastic net (EN) regularization, sparse partial least squares (PLS) - discriminant analysis (SPLS-DA), and sparse generalized PLS (SGPLS). Cook’s distance (Cook’s D) is used to evaluate the samples’ outlierness. A final consensus list of observations sorted by their outlierness level is achieved using rank product statistics corrected for multiple testing.

The three methods Rlogreg, enetLTS, and Ensemble are described in detail in section 1 of Additional File 1. For variable selection in a sparse high-dimensional setting, all three methods use the regularization of parameters. Rlogreg uses the L_1_ penalty and enetLTS uses the EN, where the regularizer is a linear combination of the L_1_ and L_2_ penalties. The EN tends to select more variables and groups of correlated variables than the L_1_ penalty. In Ensemble, the combination of L_1_ and L_2_ penalties is set in the EN, SPLS-DA, and SGPLS models. For robustness, in Rlogreg, Bootkrajang et al. [4] set label-flipping probabilities as parameters in the maximum likelihood estimator to formulate the proportion of mislabeled samples. In enetLTS, Kurnaz et al. [11, 12] applied the least trimmed square (LTS) to the EN and used the C-step algorithm to identify the optimal subset without outliers. In Ensemble, the models are performed on the original datasets with outliers, without considering robustness. In Rlogreg, the detected outliers are the misclassified observations with response *y* = 1 that are predicted as zero, or observations with *y* = 0 but predicted as 1. In enetLTS, outliers are detected using large Pearson residuals in the reweighted step. In Ensemble, Cook’s D is used to evaluate outlierness, and the consensus ranking of outlierness is achieved using the rank product test.

These three methods, which address the label error in high-dimensional data, base on different principles. Their pros and cons need to be explored so that the appropriate method can be chosen when dealing with high-dimensional datasets with mislabeled samples. Hence the accuracy of variable selection and outlier detection of these methods needs to be evaluated. The evaluation and exploration of these methods can also provide guidance for improving the results in the next step.

This article is organized as follows: In results section, results of simulation studies are presented, which were conducted to evaluate the performance of the three models, including the accuracy of variable selection, outlier detection, and prediction. The three methods were also compared by applying them to a TNBC dataset. Then the results are discussed and concluded. The simulation setting and performance measures are also described in methods section.

## Results

### Simulation results for the comparison of the three methods

In this section, we present a simulation study to investigate the performance of Rlogreg, Ensemble, and enetLTS. The accuracy of variable selection, outlier identification, and prediction, and the running time of the three methods are compared in scenarios with various sample sizes, dimensions, and proportions of outliers. Details of the simulation setting and performance measures are presented in the last section.

**Fig. 1:**
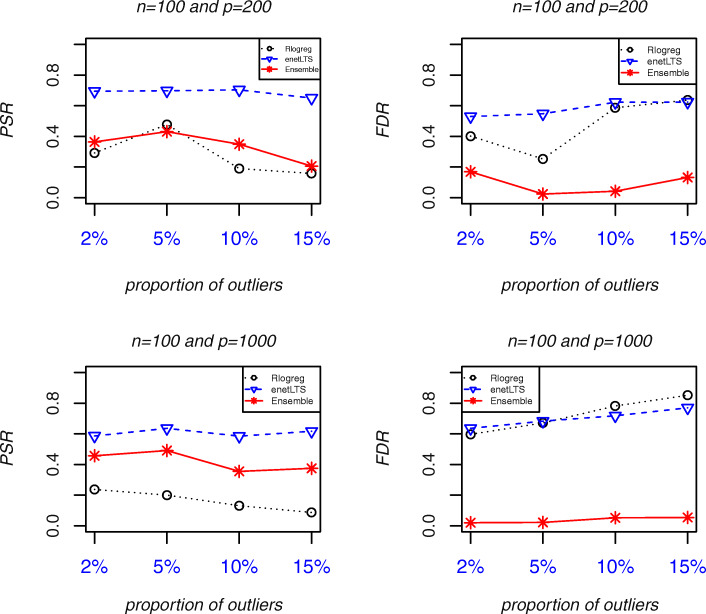
Variable selection accuracy of Rlogreg, enetLTS, and Ensemble when *n* = 100. **Abbreviations**: *PSR*, Positive Selection Rate. *FDR*, False Discovery Rate

**Fig. 2:**
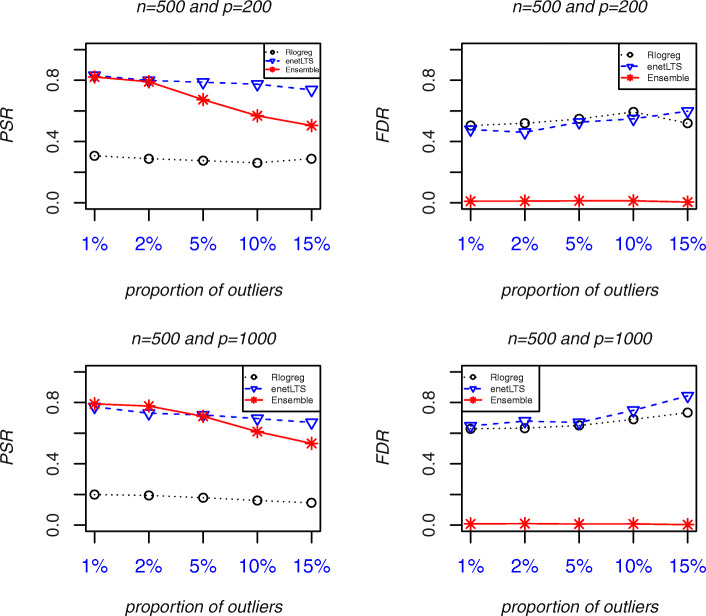
Variable selection accuracy of Rlogreg, enetLTS, and Ensemble when *n* = 500. **Abbreviations**: *PSR*, Positive Selection Rate. *FDR*, False Discovery Rate.

**Fig. 3:**
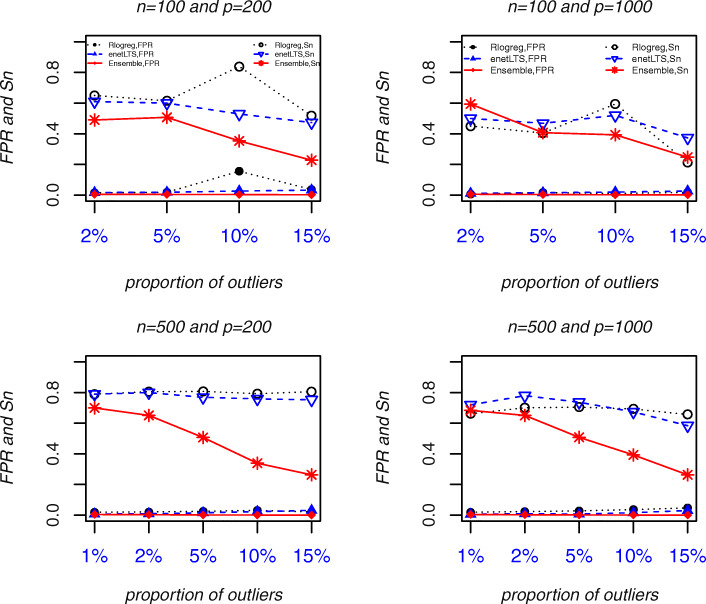
Outlier detection accuracy of Rlogreg, enetLTS, and Ensemble. Abbreviations: *Sn*, sensitivity. *FPR*, False Positive Rate

**Fig. 4:**
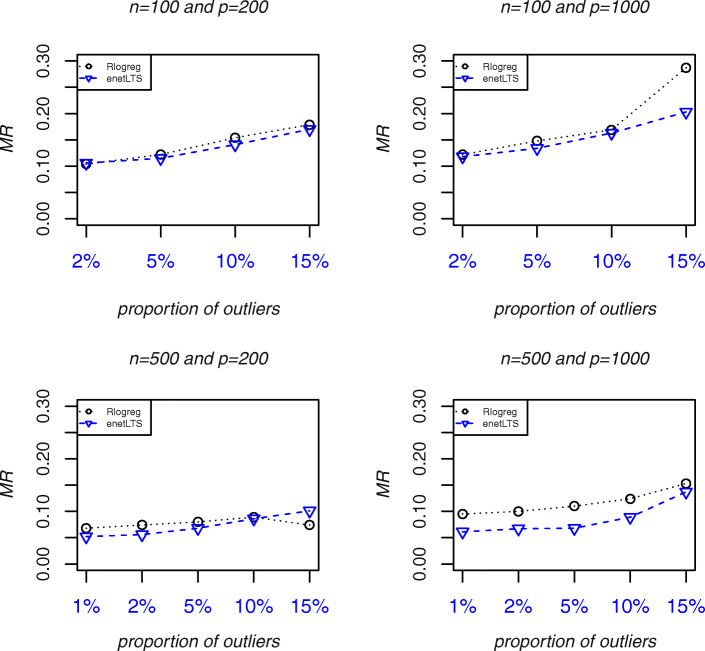
Prediction accuracy of Rlogreg, enetLTS. Abbreviations: *MR*, Misclassification Rate

**Fig. 5:**
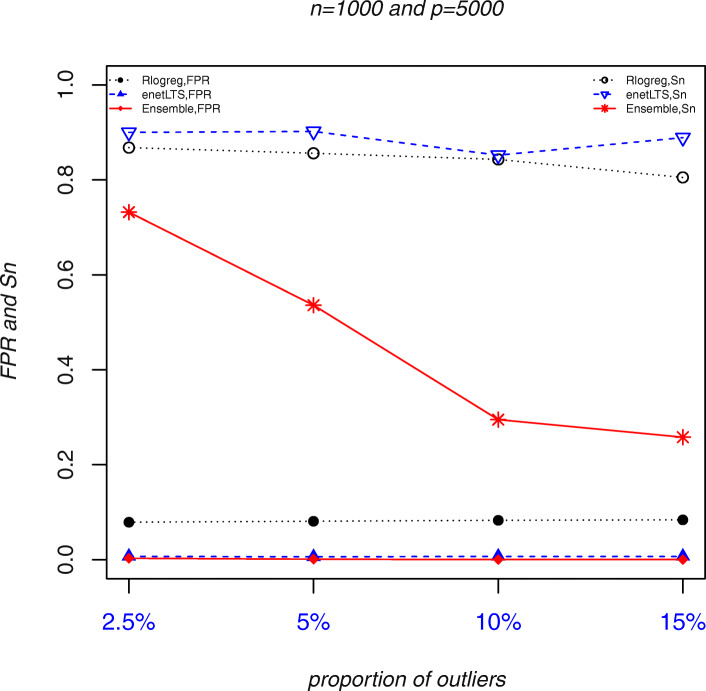
Outlier detection accuracy for the simulated datasets based on the TNBC dataset. Abbreviations: *Sn*, sensitivity. *FPR*, False Positive Rate

The results of running the three methods on simulated datasets are shown in Figs. 1–5. Section S2 (Table S2–1 to S2–4) of Additional File 1 gives the results in more detail, including the accuracy of variable selection, outlier detection, and prediction.

The high-dimensional variables were selected using indicators that represent the variable selection accuracy, namely positive selection rate (*PSR*) and false discovery rate (*FDR*) [13]. *PSR* indicates the proportion of real disease-related biomarkers that are screened out, and *FDR* indicates the proportion of biomarkers screened out that are not related to the disease. We used a comprehensive indicator *GM* [13, 14], which is the geometric mean of (*PSR* and (1 − *FDR*)) to measure the accuracy of variable selection. High *PSR*s and low *FDR*s will give high *GM*s, which indicates high accuracy of variable selection.

The *PSR*s and *FDR*s of the three methods when *n* = 100 and *p* = 200 and 1000 are shown in Fig. 1. Ensemble had the lowest *FDR* among the three methods, and the Ensemble *PSR* was lower than the enetLTS *PSR*. The *PSR* and *FDR* of enetLTS were both high. Rlogreg had the lowest *PSR* among the three methods, and the *FDR* was high. The *GM*s indicated that Ensemble had the highest variable selection accuracy, followed by enetLTS, then Rlogreg with the lowest variable selection accuracy. The Ensemble *PSR*s decreased when the proportion of outliers increased, whereas the enetLTS *PSR*s did not change much when the proportion of outliers increased.

The *PSR*s and *FDR*s of the three methods when *n* = 500 and *p* = 200 and 1000 are shown in Fig. 2. When the sample size was increased from *n* = 100 to n = 500, the variable selection accuracy improved for all three methods, but increased the most for Ensemble. When the proportion of outliers was ≤5%, the Ensemble *PSR* was close to the enetLTS *PSR* (approximately 0.8) and the Ensemble *FDR* was very low (about 0.01) but the enetLTS *FDR* was much higher. These results show that with ≤5% outliers, the Ensemble variable selection accuracy was very high. However, with 10 and 15% outliers, the Ensemble *PSR*s decreased to about 0.6 and 0.5, respectively, whereas the enetLTS *PSR*s decreased very little with the higher proportions of outliers; with 15% outliers, the enetLTS *PSR* was about 0.7. The Rlogreg *FDR* was similar to the enetLTS *FDR*, but the Rlogreg *PSR* was much lower than the enetLTS *PSR*, so the Rlogreg variable selection accuracy was the lowest among the three methods.

These results show that Ensemble had the highest variable selection accuracy, as measured by the *GM*, among the three methods, mainly because its *FDR* was much lower than the *FDR*s of the other two methods. The Ensemble variable selection accuracy was better with the large sample size. When the sample size was *n* = 500 and the proportion of outliers was ≤5%, the Ensemble and enetLTS *PSR*s were similar. However, when the proportion of outliers was 10% or 15%, the Ensemble and enetLTS *PSR*s were quite different, which implies that Ensemble may miss some variables that affect the response variables whereas the enetLTS *PSR*s decreased very little.

The outlier detection accuracy of the three methods is shown in Fig. 3. Here we used two indicators *Sn* (sensitivity) and *FPR* (false positive rate) in the screening test [13]. The outliers to be identified is regarded as patients to be detected in the screening test. *Sn* represents the proportion of truly misclassified outliers among outliers that identified. *FPR* represents the proportion of correctly labeled samples that are determined to be misclassified. The outliers identified by enetLTS and RLogreg had the highest *Sn*, but the Rlogreg *FPR*s were higher (and > 0.05 in some cases) than the enetLTS *FPR*s, which were all within 0.05. Ensemble has the lowest *Sn* and *FPR*s among the three methods. When the proportion of outliers was 1% or 2%, the Ensemble and enetLTS *Sn*s were similar, but with ≥5% outliers, the differences between the *Sn*s began to increase. With 15% outliers, the Ensemble *Sn* dropped to about 0.25. When the sample size was *n* = 500, the enetLTS *Sn* increased by 10 to 20% compared with its value for the smaller sample size (*n* = 100). The enetLTS *Sn* decreased slightly with the higher proportions of outliers, but the decrease was relatively small.

These results show that, overall, enetLTS had the highest outlier detection accuracy among the three methods. The enetLTS *Sn* was high and the *FPR*s were all within 0.05, even when the proportion of outliers was relatively large. With 1% or 2% outliers, Ensemble had high outlier detection accuracy, but with higher proportions of outliers many outliers were missed. Although the RLogreg *Sn* was high, the *FPRs* also were high and sometimes exceeded 5%.

The prediction accuracy of the three methods is shown in Fig. 4. Their predictive performances were evaluated by calculating the misclassification rate (*MR*) from test datasets without outliers. The enetLTS *MR* was lower than the Rlogreg *MR* in most cases. Because the Ensemble variables are the intersection of variables selected by the three models EN, SPLS-DA, and SGPLS, the Ensemble *MR* could not be computed.

The leverage point refers to the outlier in the independent variable space; for example, when the gene expression data of a sample deviates from the gene expression data of most samples. We mainly examine the effect of misclassified observations on the various methods if their independent variables deviate from most observations. Because gene expression data are quality controlled before they are analyzed, none of the samples will deviate significantly. The degree of deviation was set as 3, that is, the independent variables of wrongly mislabeled samples follow independent normal distribution *N* (3,1). The results of these three methods with the simulated data were similar with and without leverage. Table S2–6 of Additional File 1 gives the results in more detail.

To make the scenario closer to real data, we set up a simulated dataset based on the triple-negative breast cancer (TNBC) dataset [7], which is publicly available from The Cancer Genome Atlas (TCGA) Data Portal. The simulated dataset had a sample size of *n =* 1000, among which 500 samples had y values of 1 (TNBC group) and 500 samples had y values of 0 (non-TNBC group). A subset with *p* = 5000 was selected randomly from the TNBC dataset. The mean vector and covariance matrix for the TNBC and the non-TNBC groups were obtained from the corresponding subsets, and then the normally distributed random variables were generated.

We randomly selected samples and changed the labels to obtain the misclassified samples. The proportions of misclassified samples were set as 2.5, 5, 10, and 15%. The ability of the three methods to identify outliers is shown in Fig. 5 and Additional File 1: Tables S2–5.

The outlier detection accuracy was highest for enetLTS with *FPR*s of < 1% and the highest *Sn* among the three methods, as shown in Fig. 5. Although the Rlogreg *Sn* was close to that of enetLTS, its *FPR* of about 8% was much higher than the enetLTS *FPR*. The Ensemble *FPR* was very low (close to 0), but its *Sn* was low, especially when the proportion of outliers was large; with 10% or 15% outliers, the *Sn* was < 30%.

The results show that, when the proportion of outliers was ≤5%, the outlier detection accuracy was highest for Ensemble. However, with 10% or 15% outliers, although the overall *GM* still showed that Ensemble had the highest accuracy, the Ensemble *PSR* was lower than the enetLTS *PSR*, and some variables that affect the dependent variable were missed. With even higher proportions of outliers, the difference between these two methods would further increase. These results show that enetLTS had the highest outlier detection accuracy with *FPR*s < 0.05 and the highest *Sn* in most cases. The prediction accuracy of enetLTS was higher than that of Rlogreg.

### Combining two methods to improve the accuracy of variable selection

When the proportion of outliers was small, Ensemble had high accuracy of variable selection; however, when the proportion of outliers was large, its accuracy was greatly reduced. Conversely, regardless of the proportion of outliers, enetLTS had the highest outlier detection accuracy among the three methods.

Considering the advantages and disadvantages of the two, we combine the two methods. That is, when the proportion of outliers is large, enetLTS was used first to identify the outliers. Then Ensemble was applied on the subset with outliers removed. Because the proportion of misclassified samples in the subset will be smaller, the accuracy of Ensemble’s variable selection will increase in this case.

With 15% outliers, enetLTS was used to identify the outliers, than Ensemble was run on the subset. The Ensemble *PSR* increased from 0.533 to 0.644, and the *GM* increased from 0.714 to 0.786 as a result. The results are shown in Table 1.

**Table 1:** Results of Ensemble for the datasets with *n* = 500, *p* = 1,000, ε = 0.15

data	*Model size*	*PSR*	*FDR*	*GM*
Original data	16.06	0.533	0.003	0.714
Subset^*^	19.79	0.644	0.022	0.786

*: This subset is the universal data set after removing outliers identified by enetLTS

### The computation times of the three methods

The computation times of the three methods are summarized in Table 2. The computations were performed on an Intel Core i7-6500 U @2.50GHz processor. The CPU time was reported in seconds as an average over five repetitions. From Table 3, the computation time of Rlogreg was considerably lower than that of the other two methods because regularization parameterλwas determined using Bayesian regularization, which saved the time that cross-validation would take. The regularization parameters of Ensemble and enetLTS were both resolved by cross-validation. However, enetLTS required much more time because cross-validation was conducted at each iterative step of the C-step algorithm.

**Table 2:** The computation times of the three methods for the datasets with *n* = 500, *p* = 1,000, and ε =0.1

*Methods*	*Mean(s)*
Rlogreg	27.20
enetLTS	6489.06
ensemble	387.89

**Table 3:** Number of outliers detected and genes identified using the three methods for the TNBC dataset

*Methods*	*Model size*	*Number of outliers detected*
Rlogreg	32	109
enetLTS	433	68
Ensemble	5	30

### Results of the analysis on a TNBC dataset

We compared the application of the three methods on a TNBC dataset from the TCGA-BRCA data collection. The BRCA RNA-Seq fragments per kilobase per million (FPKM) dataset was imported using the ‘brca.data’ R package (https://github.com/averissimo/brca.data/releases/download/1.0/brca.data_1.0.tar.gz).

A total of *n* = 1019 patients with solid tumors and 19,688 genes, including the three key TNBC-associated genes, estrogen receptor (ER), progesterone receptor (PR), and human epidermal growth factor receptor 2 (HER2), were considered for further analysis. In consideration of the impact of possible confounding, like Lopes, et al. [7], we considered the inclusion of two variables, age and ethnicity, which are statistically significant through univariate Logisitc regression, and the missing observations in these two variables were removed. Finally, a total of 924 samples and 19690variables were included.

The TNBC response variable Y was created based on the clinical variables ER and PR as detected by immunohistochemistry (IHC), and HER2 as detected by IHC and/or fluorescence in situ hybridization (FISH); Y was “1” (TNBC) when ER, PR, and HER2 were negative and “0” when at least one of the three variables was positive. There were three variables for HER2: HER2 (IHC) level, HER2 (IHC), and HER2 (FISH). The values of IHC level were “0” (negative), “1+” (negative), “2+” (indeterminate), and “3+” (positive); the values of IHC status were “equivocal”, “indeterminate”, “negative”, and “positive”. Thirteen individuals had discrepant labels for HER2 (IHC) level and HER2 (IHC) status, and 15 cases had inconsistent labels in HER2 (IHC) and HER2 (FISH). These individuals were potential outliers and were referred to as “suspect individuals” by Lopes et al. [7]. We checked whether the outliers detected by the three methods included these suspect individuals.

The distributions of the FPKM values for ER, PR, and HER2 in the TNBC and non-TNBC groups are presented in Table 4.

**Table 4:** Summary of FPKM values obtained for ER, PR and HER2 for the individuals under study

	Class	Min.	1stQu	Median	Mean	3rdQu	Max
ER	0	0.016	16.144	36.667	47.881	69.649	272.203
	1	0.019	0.160	0.351	1.530	0.828	29.979
PR	0	0.008	0.600	4.228	12.012	15.326	327.913
	1	0.001	0.040	0.079	0.712	0.186	22.978
HER2	0	0.605	26.580	38.732	99.741	58.801	1668.353
	1	1.561	13.964	19.776	21.991	26.058	103.68

To analyze the TNBC dataset, for Rlogreg, the initial setting of the Γ matrix was $$ \left(\begin{array}{cc}0.925& 0.075\\ {}0.075& 0.925\end{array}\right) $$ and, for Ensemble and enetLTS, the parameters were set in accordance with the settings used with the simulated data.

Rlogreg detected 109 outliers, all of which were non-TNBCs. They were ranked from high to low according to the absolute values of the Pearson residuals and the top 20 outliers are listed in Table 5. The ER, PR, and HER2 genes in the 109 predicted non-TNBC patients all had low expression values, which indicated they should have been classified as TNBC patients. For example, individual “TCGA-AN-A0FJ” had different HER2 labels (positive by IHC status and negative by IHC level) and was classified as non-TNBC; however, the low HER2 (14.28), ER (0.08), and PR (0.04) expression values indicated that this individual was more likely to be a TNBC patient. Similarly, individual “TCGA-AN-A0FX” was labeled positive by IHC status and negative by IHC level and was classified as non-TNBC; however, the HER2 (24.02), ER (0.08), and PR (0.04) expression values indicated that this individual might be a TNBC patient. Individual “TCGA-LL-A5YP” also was classified as non-TNBC but had discordant HER2 labels and HER2 (15.10), ER (0.16), and PR (0.05) expression values, which indicated that the individual was more likely to be a TNBC patient. Among the 109 outliers detected by Rlogreg, there were nine suspect individuals with discordant HER2 labels.

**Table 5:** Top 20 outliers detected using Rlogreg for the TNBC dataset^*^

ID	Rank	ER	PR	HER2	y	HER2	HER2	HER2	Perres^**^
						level	_status	_FISH	
TCGA-A8-A07U	1	1.74 (−)	0.21 (+)	31.96	non-TNBC		–	–	1.36
TCGA-E9-A22G	2	0.44 (−)	0.02 (−)	15.32	non-TNBC		+		1.32
**TCGA-AN-A0FJ**	**3**	**0.08 (+)**	**0.04 (−)**	**14.28**	**non-TNBC**	**1+**	**+**		**1.29**
TCGA-AR-A251	4	1.57 (+)	0.10 (−)	14.02	non-TNBC	2+	Equiv	–	1.23
TCGA-BH-A5IZ	5	5.12(+)	0.03(−)	28.08	non-TNBC		–	–	1.22
TCGA-D8-A1XW	6	0.32 (−)	0.11 (+)	21.03	non-TNBC	1+	–		1.22
TCGA-AR-A1AJ	7	1.47(+)	0.07(−)	9.74	non-TNBC		–		1.19
TCGA-D8-A1JM	8	5.00(+)	0.008(−)	21.85	non-TNBC	1+	–		1.19
TCGA-E2-A1II	9	0.14(−)	0.19(+)	10.73	non-TNBC	1+	–		1.18
TCGA-A8-A07R	10	0.07 (−)	0.02 (−)	28.53	non-TNBC	2+	+		1.17
TCGA-A7-A13E	11	0.82 (+)	0.06(−)	46.08	non-TNBC	2+	Equiv	–	1.17
TCGA-B6-A0IJ	12	1.18(+)	0.56(+)	11.12	non-TNBC				1.17
TCGA-A2-A1G1	13	0.53 (−)	0.17 (−)	819.76	non-TNBC	2+	Equiv	+	1.16
TCGA-A2-A0YJ	14	0.09 (+)	0.03(−)	240.24	non-TNBC	0	–		1.15
TCGA-AO-A0JL	15	0.63 (−)	0.08(−)	63.6	non-TNBC	1+	–	+	1.15
**TCGA-AN-A0FX**	**16**	**1.13(−)**	**0.64(−)**	**24.02**	**non-TNBC**	**1+**	**+**		**1.15**
TCGA-AR-A1AH	17	0.03 (+)	0.03 (−)	34.12	non-TNBC		–		1.14
TCGA-LL-A6FR	18	0.33(−)	0.044(+)	32.23	non-TNBC	2+	Equiv	+	1.12
**TCGA-LL-A5YP**	**19**	**0.16(+)**	**0.051(−)**	**15.09**	**non-TNBC**	**1+**	**–**	**+**	**1.11**
TCGA-A7-A13D	20	0.52 (−)	0.81 (+)	42.28	non-TNBC	2+	Equiv	–	1.07

*:including the expression values, IHC, and FISH tests of ER, PR, and HER2(individuals highlighted in bold are suspect individuals). **: Rank, the rank of outlierness by the abstract value of Pearson residual. Perres, the abstract value of Pearson residual

The relatively low HER2, ER, and PR expression values of the individuals who were ranked 3, 4, 5, 7, 12, 16, and 19 indicated they were more likely to be TNBC patients, whereas the very high HER2 expression values (819.76 and 240.24) of the individuals who were ranked 13 and 14 indicated they were likely labeled correctly as non-TNBC. These results suggest that Rlogreg may produce false positives for outlier detection.

EnetLTS detected 68 outliers and 433 associated genes as shown in Tables S4–1 and Table S4–6 of Additional File 1, respectively. The 68 outliers included 3 TNBC and 65 non-TNBC individuals. The top 20 outliers with the highest Pearson residuals are listed in Table 6. Seven of the 68 outliers were suspect individuals with inconsistent HER2 labels. The HER2, ER, and PR expression values for suspect individuals “TCGA-A2-A04U”, “TCGA-AN-A0FX”, “TCGA-LL-A5YP”, and TCGA-AN-A0FJ were relatively low, which indicated they were more likely to be TNBC patients.

**Table 6:** Top 20 outliers detected using enetLTS for the TNBC dataset^*^

ID	ER	PR	HER2	HER2_level	HER2_status	HER2_FISH	y	Perres^**^
TCGA-E9-A22G	0.44 (−)	0.02 (−)	15.32		+		non-TNBC	37.22
TCGA-A2-A0YJ	0.09 (+)	0.03 (−)	240.24	0	–		non-TNBC	35.66
TCGA-A7-A13E	0.82 (+)	0.06 (−)	46.08	2+	Equiv	–	non-TNBC	33.23
**TCGA-A2-A04U**	**0.02 (−)**	**0.02 (−)**	**9.64**	**1+**	**–**	**+**	**non-TNBC**	**32.24**
TCGA-AR-A0TP	0.04 (+)	0.03 (−)	13.39		–		non-TNBC	32.15
**TCGA-AN-A0FX**	**1.13 (−)**	**0.64 (−)**	**24.02**	**1+**	**+**		**non-TNBC**	**30.70**
TCGA-AR-A251	1.57 (+)	0.10 (−)	14.02	2+	Equiv	–	non-TNBC	30.23
TCGA-LL-A6FR	0.33 (−)	0.04 (+)	32.13	2+	Equiv	+	non-TNBC	30.12
TCGA-AC-A62X	0.19 (+)	0.02 (−)	28.53				**non-TNBC**	30.11
TCGA-BH-A5IZ	5.12 (+)	0.03 (−)	28.08		–	–	non-TNBC	29.99
TCGA-OL-A5S0	0.09 (+)	0.06 (−)	31.92			+	non-TNBC	29.50
**TCGA-LL-A5YP**	**0.16 (+)**	**0.05 (−)**	**15.10**	**1+**	**–**	**+**	**non-TNBC**	**29.05**
TCGA-LL-A8F5	1.08 (+)	0.04 (−)	11.86	1+	–		non-TNBC	27.77
TCGA-B6-A0IJ	1.18 (+)	0.46 (+)	11.12				non-TNBC	27.74
TCGA-A7-A13D	0.52 (−)	0.81 (+)	42.28	2+	Equiv	–	non-TNBC	27.59
TCGA-AR-A24Q	1.00 (+)	0.36 (−)	20.67		–		non-TNBC	27.44
TCGA-S3-AA0Z	16.67 (+)	0.07 (+)	33.07	1+	Equiv	–	non-TNBC	27.31
**TCGA-AN-A0FJ**	**0.08 (+)**	**0.04 (−)**	**14.28**	**1+**	**+**		**non-TNBC**	**27.29**
TCGA-AR-A1AH	0.03 (+)	0.03 (−)	34.12		–		non-TNBC	27.04
TCGA-D8-A1JM	5.00 (+)	0.01 (−)	21.85	1+	–		non-TNBC	26.93

*:including the expression values, IHC, and FISH tests of ER, PR, and HER2(individuals highlighted in bold are suspect individuals.). **:Perres, the abstract value of Pearson residual

Many outliers, including individuals “TCGA-E9-A22G”, “TCGA-AR-A0TP”, “TCGA-AR-A251”, and “TCGA-B6-A0IJ”, were labeled as non-TNBC patients, but the low expression values of the three genes indicated they were more likely to be TNBC patients. However, some outliers, including individuals “TCGA-A2-A0YJ”, “TCGA-A7-A13E”, and “TCGA-A7-A13D”, had high expression values for one or more of the three genes, which indicated they were likely labeled correctly as non-TNBC patients.

Ensemble identified 30 outliers and 5 genes. Ten patients with TNBC and 20 patients with non TNBC were found in 30 abnormal patients. Table 7 lists the 20 outliers with the minimum q-values. All outliers are listed in Table S5-1 of Additional File 1.

**Table 7:** Top 20 outliers detected using Ensemble for the TNBC dataset^*^

ID	ER	PR	HER2	HER2_level	HER2_status	HER2_FISH	y	qvalues
TCGA-E9-A1ND	1.44 (−)	0.05 (−)	13.05		+		non-TNBC	8.90E-06
TCGA-AR-A1AJ	1.47 (+)	0.07 (−)	9.74		–		non-TNBC	8.46E-06
**TCGA-A2-A04U**	**0.02 (−)**	**0.02 (−)**	**9.64**	**1+**	**–**	**+**	**non-TNBC**	**1.65E-05**
TCGA-E9-A22G	0.44 (−)	0.02 (−)	15.32		+		non-TNBC	2.76E-05
TCGA-OL-A97C	16.25 (−)	8.56 (−)	24.04			–	TNBC	7.43E-05
TCGA-AC-A62X	0.19 (+)	0.02 (−)	28.53				non-TNBC	4.24E-05
TCGA-A7-A13D	0.52 (−)	0.81 (+)	42.28	2+	Equiv	–	non-TNBC	6.89E-05
TCGA-BH-A42U	9.19 (−)	1.83 (−)	38.37		–		TNBC	7.16E-05
TCGA-OL-A5S0	0.09 (+)	0.06 (−)	31.92			+	non-TNBC	7.07E-05
TCGA-E2-A1II	0.14 (−)	0.19 (+)	10.73	1+	–		non-TNBC	1.50E-04
**TCGA-A2-A0EQ**	**2.13 (−)**	**0.04 (−)**	**30.15**	**3+**	**+**	**–**	**TNBC**	**1.62E-04**
TCGA-B6-A0IJ	1.18 (+)	0.46 (+)	11.12				non-TNBC	1.55E-04
TCGA-C8-A26Y	0.12 (−)	0.05 (−)	22.92	1+	–		TNBC	1.53E-04
TCGA-A2-A1G6	23.90 (−)	21.45 (−)	29.74	1+	–		TNBC	2.24E-04
TCGA-BH-A5IZ	5.12 (+)	0.03 (−)	28.08		–	–	non-TNBC	2.18E-04
TCGA-A2-A0YJ	0.09 (+)	0.03 (−)	240.24	0	–		non-TNBC	3.42E-04
TCGA-AR-A1AH	0.03 (+)	0.03 (−)	34.12		–		non-TNBC	2.68E-04
TCGA-BH-A0DL	6.99 (+)	0.04 (−)	9.92		–		non-TNBC	3.23E-04
TCGA-E9-A1NC	0.11 (−)	0.07 (+)	15.91		+		non-TNBC	2.76E-04
TCGA-AO-A03U	0.56 (−)	0.12 (−)	17.06	0	–	–	TNBC	2.97E-04

*:, including the expression values, IHC, and FISH tests of ER, PR, and HER2(individuals highlighted in bold are suspect individuals)

There were 28 suspect individuals in the TNBC dataset. Among the 30 outliers identified by Ensemble, three were suspect individuals; among the 68 outliers detected by enetLTS, seven were suspect individuals; and among 109 outliers detected by Rlogreg, nine were suspect individuals. Because the true labels of the individuals the TNBC dataset are not known, we regarded these 28 suspect individuals as true mislabeled individuals and compared the outlier detection accuracy of three methods. we used *Sn_Ref* and *FPR_Ref* as references for the true *Sn* and *FPR*s, which is shown in Table 8. For suspect individuals, the enetLTS and Rlogreg *Sn_Ref* values were high, whereas they were low for Ensemble. However, because Rlogreg identified a large number of outliers, its *FPR*_*Ref* also was high. These results are similar to those obtained with the simulated data. Although 3% of the samples in the TNBC dataset had inconsistent labels, nearly 300 individuals were tested using only one method for the detection of HER2, and IHC was the only method used for ER and PR detection in all the individuals. Because false positives and false negatives will appear in the IHC test, the actual proportion of misclassified samples in the TNBC dataset may be higher. Therefore, *Sn_Ref* will likely underestimate the true *Sn*, and *FPR_Ref* may overestimate the actual *FPR*.

**Table 8:** Comparison of outliers detected by the three methods for the TNBC dataset

*Methods*	*Num of outliers* identified	*Sn_ref*(%)	*FPR_ref*(%)
Ensemble	30	3/28 (10.7)	27/896 (3.0)
enetLTS	68	7/28 (25.0)	36/896 (6.8)
Rlogreg	109	9/28 (32.1)	100/896 (11.2)

Notes: *Sn_Ref* and *FPR*_*ref*, which computed when the 28 suspect individuals were taken for true outliers, are as references of true sensitivity and *FPR*. *Num of outlier*, Number of outliers detected

To test the robustness of outliers selected by the three methods, 5000 genes were selected randomly from the 19,690 variables to form a random gene set. A total of 66 outliers were identified in the random gene set using enetLTS, 62 of which coincided with those in the original TNBC dataset, and seven suspect individuals with inconsistent labels also were included. A total of 33 outliers were identified in the random gene set using Ensemble, 25 coincided with those in original TNBC dataset, including four suspect individuals with inconsistent labels. A total of 125 outliers were identified in the random gene set using Rlogreg, 92 coincided with the original TNBC dataset, including nine suspect individuals with inconsistent labels. Therefore, the results for the random gene set mostly coincided with the results for the original TNBC dataset. The results for the random gene sets are described in detail in Additional File 1.

The 32 genes selected by Rlogreg are listed in Table 9. Among them, the gene encoding the fatty acid protein FABP7 was reported to be up-regulated in the TNBC dataset by [15], and elevated *FABP7* expression levels have been associated with poor prognosis. Other genes selected by Rlogreg, namely *KISS1* [16], *IGF2BP2* [17], *CALCA* [18], *PLA1A* [19], and *FAM171A1* [20], have been reported to be related to breast cancer or other types of cancer. However, the three key TNBC-associated genes ER, PR, and HER2 were not among the genes selected by Rlogreg.

**Table 9:** Genes selected by Rlogreg for the TNBC dataset

Up-regulated	UTS2(0.23), IGF2BP2(1.70), PGC(0.41), CALCA(0.17), SLC16A10(0.93), PRKAG3(0.23), PIK3CA(0.09), SEMG2(0.26), FUT5(0.21), DNMT3L(0.22), PLA1A(0.31), HIST1H2BA(0.079), FAM171A1(2.17), ADGRF1(0.26), RNF168(0.53), FABP7(0.18), TRPV6(0.49), SLC6A5(0.09), KCNJ4(0.28), FAM107A(1.03), GUSB(0.28), KISS1(0.12), C1QTNF4(0.45), OR4C5(0.24), OTOG(0.10), PRR9(0.11), PAGE3(0.24), ENSG00000273047(0.39), ENSG00000279126(0.23)
Down-regulted	IGSF11 (− 0.16),INSYN2B (− 0.14), ANHX(− 0.12)

The five genes selected by Ensemble are shown in Table 10. *ESR1* (i.e., ER), one of the three key genes of TNBC, was among them. The other four, *CA12* [21], *AGR2* [22], T*FF1* [23], and *AGR3* [24] have been reported to be up- or down-regulated in TNBC.

**Table 10:** Genes selected by Ensemble for the TNBC dataset

CA12, ESR1, AGR2, TFF1, AGR3	

A total of 433 genes were selected by enetLTS. The 40 genes with the largest absolute value of the coefficient are listed in Table 11 and details of all the selected genes are provided in Table S4–6 Additional File 1. The five genes selected by Ensemble were among the 433 genes. Two key genes of TNBC, ER and PR, were among the genes selected by enetLTS. Other genes selected by enetLTS, *FOXA1* [25], *GATA3* [25], *SPDEF* [26], *FOXC1* [27], *EN1* [28], *HORMAD1* [29], *KRT16* [30], and *CT83* [31], have been reported to be related to TNBC.

**Table 11:** Top 40 Genes selected by enetLTS for the TNBC dataset

Up-regulated	VGLL1(0.245), PPP1R14C(0.232), RGMA(0.213), CT83(0.207), EN1(0.184), UGT8(0.182), FOXC1(0.178), SMOC1(0.173), HORMAD1(0.17), SFT2D2(0.161), CA12(− 0.155), C19orf47(0.147), MSLN(0.138), PRSS16(0.129), RCOR2(0.12), FGD1(0.12), MIA(0.119), FAM171A1(0.116), KRT16(0.115), KRT23(0.111), KCNK5(0.109), COL9A3(0.097), TMSB15A(0.096)
Down-regulted	FOXA1(− 0.276), SPDEF(− 0.264), GPR160(− 0.197), GATA3(− 0.171), CA12(− 0.155), CAPN13(− 0.143), DNALI1(− 0.142), SLC40A1(− 0.135), AGR2(− 0.129), TFF3(− 0.121), RND1(− 0.118), PGAP3(− 0.11), SNCG(− 0.11)HSPB8(− 0.108), SLC44A4(− 0.1), SIDT1(− 0.097), MMP19(− 0.094), AGR3(− 0.093)

We did not know the true genes associated with TNBC, nor the true outliers in the TNBC dataset. The results showed that when the proportion of outliers was > 5%, the Ensemble *PSR* was lower than the enetLTS *PSR*, and the larger the proportion of outliers, the greater was the gap between the two. Ensemble selected only five genes, and some genes that have been reported to be related to TNBC were missed, probably because of the relatively large proportion of misclassified samples. Although only 3% of the samples in this study had inconsistent labels, nearly 300 individuals were tested using only one method to detect HER2, and only one method, IHC, was used to detect ER and PR in all the individuals in the TNBC dataset. It has been reported that up to 20% of IHC test for ER and PR worldwide might be inaccurate (false negative or false positive), mainly due to variations in preanalytic variables, thresholds for positivity, and interpretation criteria [32]. Therefore, there may be more misclassified individuals in the TNBC dataset.

The results showed that when the proportion of outliers was relatively large, enetLTS had high outlier detection accuracy, and when the proportion of outliers was low, Ensemble had high variable selection accuracy. We combined the advantages of these two methods and removed 68 outliers identified by enetLTS, then run Ensemble on a subset of 856 samples, which further improved the accuracy of gene selection.

**Fig. 6:**
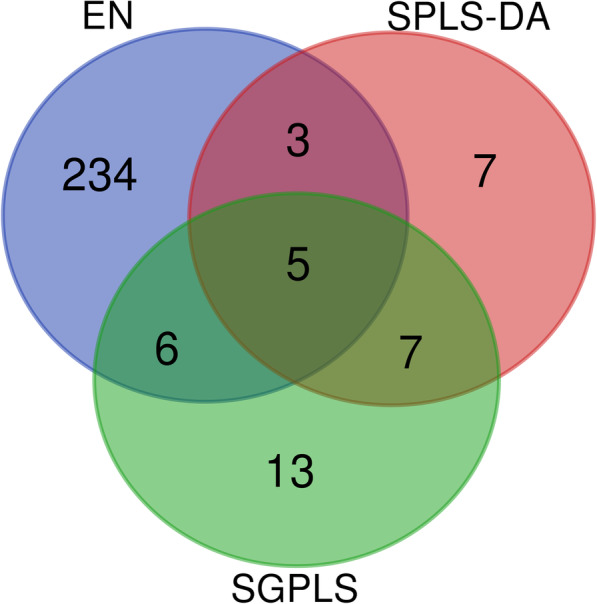
The intersection of genes selected by Ensemble’s three models on the original TNBC dataset

**Fig. 7:**
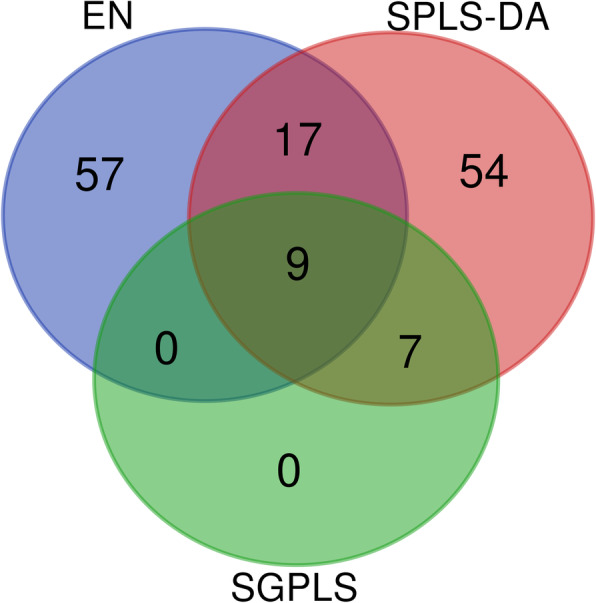
The intersection of genes selected by Ensemble’s three methods on the subset with outliers removed

The prediction index *MR* of the three models in Ensemble was much lower on the TNBC subset with 68 outliers removed than it was on the original TNBC dataset as shown in Table 12; the EN *MR* decreased from 0.012 to 0, the SPLS-DA *MR* decreased from 0.064 to 0.008, and the SGPLS *MR* decreased from 0.059 to 0.015. Figures 6 and 7 show the intersection of the three Ensemble models for screening genes on the original TNBC dataset and on the subset with outliers removed. With the subset, all the genes screened by SGPLS overlapped with the genes screened by the other two models. The intersection of the genes screened by EN and SPLS-DA also increased from eight in the original TNBC dataset to 26 in the subset. These results show that the consistency of gene screened by the three Ensemble models was greatly increased after removing outliers.

**Table 12:** Results of Ensemble three models for the original TNBC data and subset with outliers removed

***Dataset***	***EN***		***SPLS-DA***		***SGPLS***	
	Model size**	***MR#***	***Model size***	***MR***	***Model***	***MR***
Original data	248	0.012	22	0.064	31	0.059
Subset*	83	0.000	87	0.008	16	0.015

*: This subset is the original data set after removing 68 outliers identified by enetLTS.**: Model size, number of variables. #:MR, Misclassification Rate

The intersection of variables selected using the three Ensemble models increased from five to nine genes, namely *CA12* [9], *GABRP* [33], *VGLL1* [34], *AGR2* [35], *GATA3* [25], *FOXA1* [25], *TFF3* [36], *AGR3* [24], and *KRT16* [30] in Table 13, all of which have been reported to be related to TNBC.

**Table 13:** Genes selected by Ensemble for the TNBC subet^*^

CA12,GABRP, VGLL1, AGR2, GATA3, FOXA1, TFF3, AGR3, KRT16	

*: This subset is the original data set after removing 68 outliers identified by enetLTS

Because the prediction accuracy of the three Ensemble models was very high, the genes selected by pairs of models were also listed in Table 14. Among the genes selected by both EN and SPLS-DA, *ESR1*, one of three key variables, and *PHGDH* [37], *RARRES1* [38], *SPDEF* [26], *PSAT1* [37], and *FABP7* [15] have been reported to be related to TNBC. Among the genes selected by both SPLS-DA and SGPLS, *SLPI* [39], *TFF1* [23], and *KRT6B* [40] have been reported to be related to TNBC. Other selected genes, *SLC40A1* [41], *ADAMTS15* [42], *THSD4* [43], *GREB1* [44], and *SLC44A4* [45] have been reported to be related to breast cancer, and *ZG16B* [46], *FDCSP* [47], and *SRARP* [48] have been associated with other types of tumors. The correlation between these genes and TNBC should be verified by further experiments.

**Table 14:** Intersection of genes selected by two methods of Ensemble for the TNBC subset^*^

EN and SPLS-DA	ESR1, PHGDH, RARRES1, PI3, SPDEF, PSAT1, SLC40A1, ZG16B, CMBL, FABP7, ADAMTS15, PRR15, SRARP, THSD4, GREB1, PPP1R14C, SLC44A4
SPLS and SGPLS	SLPI, TFF1, CALML5, FDCSP, KRT6B, KRT81, age

*: This subset is the original data set after removing 68 outliers identified by enetLTS. After removing the intersection of the three methods, outliers identified by EN and SGPLS had no intersection

## Discussion

Mislabeled samples in omics data lead to two main problems: how to identify associated biomarkers accurately and avoid the influence of mislabeled samples, and how to detect mislabeled samples accurately.

Rlogreg had the lowest variable selection accuracy among the three methods tested, and lower outlier identification accuracy than enetLTS. The computation time for Rlogreg was considerably lower than those of the other two methods because the regularization parameter *λ* is determined using Bayesian regularization, which is faster than cross-validation. However, this way of determining *λ* affected the accuracy of variable selection, which was worse than that of enetLTS, which uses cross-validation to determine the regularization parameter. Additionally, because Bootkrajang et al. [4] set the misclassified samples predicted by Rlogreg as outliers, many outliers were detected and the *FPR* was high.

Ensemble and enetLTS both use cross-validation to determine the regularization parameters. However, enetLTS required much more time than Ensemble because cross-validation is conducted at each iterative step of the C-step algorithm in enetLTS. For outlier detection, individuals with significant Pearson residuals more than Φ(0.9875) were regarded as outliers in enetLTS. The outlier detection accuracy of enetLTS was better than that of Rlogreg, and enetLTS was stable when the proportion of outliers increased. Least trimmed square (LTS) is an effective method to solve the masking phenomenon in which many outliers are located close together in a low-dimensional dataset [49]. The results showed that enetLTS also was an effective method to detect outliers in a high-dimensional dataset. For variable selection, enetLTS is equivalent to EN when EN was applied on the subset with outliers removed. EN tends to include all relevant variables, so more variables are selected, resulting in high *PSR*s and *FDR*s for enetLTS. We ran Ensemble on the TNBC dataset after removing the outliers identified by enetLTS. And one of the three ways that Ensemble includes is EN. Therefore, using Ensemble is equivalent to running three methods on this subset, so by keeping the variables with strong correlation with dependent variables through their intersection, the *FDR* can be reduced.

The EN model in Ensemble also was compared with enetLTS by Kurnaz et al. [11]. The results showed that on contaminated data, enetLTS performed better than the EN for variable selection with a lower *FPR* and better precision of coefficients. However, associated genes with substantial effects were not influenced by outliers and were detected by all three Ensemble models. Then, by finding the intersection of variables selected by the three Ensemble models, the genes with the strongest effects are selected. Because the intersection contained few genes, Ensemble had a very low *FDR* and a lower *PSR* than enetLTS. When the proportion of outliers was relatively large, we found that the variable selection accuracy of Ensemble decreased, especially the *PSR*. This may be because, without considering robustness, when the original datasets with outliers were used, the variable selection of the three Ensemble models may have been influenced by the outliers. EnetLTS performs the EN estimation after removing outliers, so the enetLTS *PSR* is less affected by the outliers.

All the results showed that the variable selection accuracy of Ensemble on high-dimensional data was high. Ensemble is a way of modeling using a variety of different basic models, so, as long as the basic model has diversity and independence, Ensemble will usually have low error rates. Many researches on Ensemble models have combined multiple machine learning methods to improve the accuracy of predictions [50]. Because the different models “look” at the data from different angles, they are not flexible enough to make changes in the training set to more accurately summarize new data and improve the generalization ability of the model. Rather, the Ensemble approach aims to seek the wisdom of the group to build a model that is closer to reality [51].

Overall, the outlier detection accuracy of Ensemble was worse than that of enetLTS. Ensemble achieved consensus with rank product statistics corrected by multiple testing, which led to fewer outliers detected by Ensemble and a lower *Sn* than enetLTS. Further, Cook’s D derived from EN, SPLS-DA, or SGPLS may have been influenced by outliers. Conversely, the Pearson residual of enetLTS was derived from the subset without outliers, which may explain why enetLTS detected more outliers with a higher *Sn* than Ensemble.

When the proportion of outliers was relatively low, such as ≤5%, the results showed that Ensemble had high variable selection accuracy. Although none of the three Ensemble models considered robustness, they were less affected by the proportion of outliers, so the *FDR* of variable selection was reduced by intersection. When the proportion of outliers was large, such as > 5%, the results showed that using enetLTS first to identify outliers and then using Ensemble improved the overall variable selection accuracy. In practice, the proportion of outliers can be determined according to the inaccuracy rate of the diagnostic methods used; for example, the inaccuracy rate of IHC detection is about 20%. EnetLTS is the recommended method for the identification of misclassified samples regardless of the proportion of outliers in a dataset.

The identified misclassified samples need to be further checked using more accurate tests or multiple tests so that experimental or diagnostic errors can be corrected to avoid subsequent treatment failure caused by the wrong treatment. If, after verification, the identified misclassified samples were found not to be caused by such errors, it may mean that the disease classification of these samples had different response patterns compared to their covariate combinations. Taking the TNBC data for example, if the identified misclassified sample is labeled TNBC, and it is not a diagnostic error after verification, it indicates he/she should be labeled non-TNBC based on the genes screened from the vast majority of individuals. For these heterogeneous samples, we suggest that further analysis can be done in this way. The propensity score [52] can be used to match these heterogeneous TNBC individuals according to the gene expression values of genes (screened from the vast majority of individuals) among non-TNBC individuals. In this way, specific genes related to TNBC can be found in these heterogeneous TNBC individuals, and these specific genes are different from associated genes screened from the vast majority of individuals. Further research may find suitable individualized treatment for these heterogeneous TNBC patients. The analysis of identified heterogeneous samples needs further research.

## Conclusions

When the proportion of outliers is relatively low and ≤ 5%, Ensemble can be used for variable selection. When the proportion of outliers is > 5%, Ensemble can be used for variable selection on a subset of data after removing outliers identified by enetLTS. For outlier identification, enetLTS is the recommended method. In practice, the proportion of outliers in a dataset can be estimated according to the inaccuracy of the diagnostic methods used.

## Methods

### Design of Simulation study

#### Simulation settings

We generated *n* = 100 and *n* = 500 observations from a *p*-dimensional multinormal distribution *N*(0, *Σ*_*p*_) with *p* = 200 and *p* = 1000. The (*i,j*) element of *Σ*_*p*_ was set to 0.9^∣*i* − *j*∣^, 1 ≤ *i*, *j* ≤ *p*. We assumed high correlation coefficients among variables because the close correlation among genes is frequently observed.

We fixed the coefficient vector as *β*^*T*^ =(1, …, 1,0, …, 0). The first 30 *β*_*i*_ were set to one and the others were set to zero. The response variable was generated according to a Bernoulli distribution with *y*_*i*_~*B*(1, *π*_*i*_), where $logit\left({\pi}_i\right)={x}_i^T\beta$ for *i = 1,2, …,n.*

We considered the following two scenarios for outliers. (1) Outliers in the response: We set $\frac{1}{3}\varepsilon$ of the observations selected randomly from the class *y*_*i*_ = 0 to one, and $\frac{2}{3}\varepsilon$ for which selected randomly from the class *y*_*i*_ = 1 to zero. Asymmetric mislabeled samples were set because they are usually more harmful and harder to be detected than symmetric ones. *ε* =0.01, 0.02, 0.05, 0.10 and 0.15 were considered. (2) Outliers in both the response and predictors: This was the same as scenario (1); however, ε of observations with outliers in the response also contained outliers in the predictors following an independent *N* (3,1) distribution.(3) To make the simulation scenario closer to the real data, we set up the simulation based on the TNBC data set . The new datasets were simulated with sample size *N* = 1000, of which 500 observations had y value of 1 (TNBC class), and 500 samples have y value of 0 (non-TNBC class). A subset with the dimension *p* = 5000 was randomly selected from the TNBC data set. The mean vector and covariance matrix corresponding to the TNBC group and the non-TNBC group were obtained respectively from the subset, and then the normally distributed random variables were generated.

For each setting mentioned above, we compared the performance of Rlogreg, Ensemble, and enetLTS. For Rlogreg, the initial gamma matrix was set to $$ \left(\begin{array}{cc}0.85& 0.15\\ {}0.15& 0.85\end{array}\right) $$, which means that the initial probability that the label was flipped from the true label one to the observed label zero, or the true label zero to the observed label one was 0.15 for both cases. Following Kurnaz et al. (2018), for enetLTS, parameter *h* was chosen as [0.75n]. Other parameters were the default settings in the R package enetLTS. Following Lopes et al. (2018), the optimization of the model parameters of EN, SPLS-DA, and SGPLS based on the mean squared error (MSE) was performed by 10-fold cross-validation.

#### Performance measures

The evaluation criteria were divided into three categories. The first category concerns the variable selection accuracy.

(1) Model size: the number of non-zero coefficients in the estimated model.

(2) Positive Selection Rate (*PSR*) and false discovery rate (*FDR*):

$$ PSR=\frac{TP}{TP+ FN}, $$

$$ FDR=\left\{\begin{array}{c}\frac{FP}{TP+ FP}, TP+ FP>0\\ {}0, TP+ FP=0\end{array}\right., $$

where true positive *TP* is the number of coefficients that are non-zero in the true model and were estimated as non-zero. In the true model, false positive *FP* represents the zero coefficients that were estimated as non-zero. False negative *FN* represents the number of non-zero coefficients that were estimated as zero. *PSR* represents the proportion of *TP* in non-zero coefficients in the actual model. Additionally, *FDR* represents the ratio of *FP* in non-zero estimated coefficients.

(3) The geometric mean of *PSR* and (1-*FDR*) (*GM*): We calculated the geometric mean of *PSR* and (1-*FDR*) to evaluate the selection performance of the methods comprehensively.

The second category of indicators evaluates the accuracy of outlier detection.

(1) The number of outliers (*Num*): Number outliers detected by a method.

(2) Sensitivity (*Sn*) and false positive rate (*FPR*):

$$ Sn=\frac{TP^{\ast }}{TP^{\ast }+{FN}^{\ast }}, $$

$$ FPR=\frac{FP^{\ast }}{FP^{\ast }+{TN}^{\ast }}, $$

where true positive TP^∗^ represents the number of actual outliers that were also detected as outliers. False positive FP^∗^ represents the number of individuals with correct labels that were detected as outliers. False negative FN^∗^ represents the number of actual outliers that were misclassified as individuals with the correct labels. True negative TN^∗^ represents the number of individuals with actual correct labels that were also identified as those with correct labels.

*Sn* represents the proportion of actual outliers that were correctly identified. *FPR* represents the proportion of individuals with correct labels that were wrongly categorized as outliers.

The third category of indicators evaluates the prediction accuracy.

(1) Misclassification rate (*MR*): *MR* represents the fraction of misclassified observations that correspond to their prediction probability by the fitted model.

We set

$$ {\hat{y}}_i=\left\{\begin{array}{cc}1& if\ {\hat{p}}_i\ge 0.5\\ {}0& if{\hat{p}}_i<0.5\end{array}\right., $$

where predicted probability ${\hat{\mathrm{p}}}_{\mathrm{i}}=\frac{\exp \left({\mathrm{x}}_{\mathrm{i}}^{\prime}\hat{\upbeta}\right)}{1+\exp \left({\mathrm{x}}_{\mathrm{i}}^{\prime}\hat{\upbeta}\right)}$ and ${\hat{\mathrm{y}}}_{\mathrm{i}}$ is the predicted response. Misclassified observations are samples with response *y* = 1 that are predicted as zero, or ones with *y* = 0 but predicted as 1.

Training data and test data were generated according to the above sampling schemes. Training data were generated to fit the model and test data to evaluate the model. The test data were generated without outliers. For each setting, we calculated the average of the performance measures over 100 simulation replicates implemented in MATLAB [53] (for Rlogreg only) and *R* software [54, 55].

## Supplementary information

**Additional file 1.**

## Data Availability

Code used in the simulation studies and for RLogreg is available on Github (https://github.com/hwsun2000/Mislabeled), which allows reproducibility. The R package enetLTS can be found at https://cran.r-project.org/web/packages/enetLTS/index.html. The code of Ensemble can be downloaded from http://web.tecnico.ulisboa.pt/susanavinga/TNBC/. The BRCA RNA-Seq FPKM dataset was imported using the ‘brca.data’ R package (https://github.com/averissimo/brca.data/releases/download/1.0/brca.data_1.0.tar.gz).

## Abbreviations

Blogreg: Bayesian logistic regression classifier
Cook’s D: Cook’s distance
EN: Elastic net
enetLTS: Robust elastic net based on the least trimmed square
*FDR*: False discovery rate
FISH: Fluorescence in situ hybridization
*FPR*: False Positive Rate
*FPR*_*ref*: As a reference of true *FPR*
*GM*: The geometric mean of *PSR* and (1-*FDR*)
IHC: Immunohistochemical
LASSO: Least absolute shrinkage and selection operator
LTS: Least Trimmed square
*MR*: Misclassification Rate
*Num*: Number of outliers
PLS: Partial least squares
*PSR*: Positive Selection Rate
Rlogreg: Sparse label-noise-robust logistic regression
*Sn*: Sensitivity
*Sn*_*Ref*: A reference of true sensitivity
SGPLS: Sparse generalized PLS
SPLS-DA: Sparse PLS - discriminant analysis
TNBC: Triple negative breast cancer

## References

[1] Zou H, Hastie T (2005). Regularization and variable selection via the elastic net. Journal of the Royal Statistical Society: Series B (Statistical Methodology).

[2] Tibshirani R: Regression shrinkage and selection via the LASSO. Journal of the Royal Statistical Society: Series B (Statistical Methodology) 1996, 58:267–288.

[3] Wold S, Ruhe A, Wold H, Dunn I (1984). WJ: **the collinearity problem in linear regression. The partial least squares (PLS) approach to generalized inverses**. SIAM J Sci Stat Comput.

[4] Bootkrajang J, Kaban A (2013). Classification of mislabelled microarrays using robust sparse logistic regression. Bioinformatics.

[5] Zhang C, Wu C, Blanzieri E, Zhou Y, Wang Y, Du W, Liang Y (2009). Methods for labeling error detection in microarrays based on the effect of data perturbation on the regression model. Bioinformatics.

[6] Khan J, Wei JS, Ringner M, Saal LH, Ladanyi M, Westermann F, Berthold F, Schwab M, Antonescu CR, Peterson C (2001). Classification and diagnostic prediction of cancers using gene expression profiling and artificial neural networks. Nat Med.

[7] Lopes MB, Verissimo A, Carrasquinha E, Casimiro S, Beerenwinkel N, Vinga S (2018). Ensemble outlier detection and gene selection in triple-negative breast cancer data. BMC bioinformatics.

[8] Wu C, Ma S (2015). A selective review of robust variable selection with applications in bioinformatics. Brief Bioinform.

[9] Segaert P, Lopes MB, Casimiro S, Vinga S, Rousseeuw PJ. Robust identification of target genes and outliers in triple-negative breast cancer data. Stat Methods Med Res. 2018;962280218794722. 10.1177/0962280218794722 PMC6745616 30146936

[10] Shevade SK, Keerthi SS (2003). A simple and efficient algorithm for gene selection using sparse logistic regression. Bioinformatics.

[11] Kurnaz FS, Hoffmann I, Filzmoser P (2018). Robust and sparse estimation methods for high dimensional linear and logistic regression. Chemometrics & Intelligent Laboratory Systems.

[12] Ternes N, Rotolo F, Michiels S. Empirical extensions of the lasso penalty to reduce the false discovery rate in high-dimensional Cox regression models. Stat Med. 2016;35(15):2561–73. 10.1002/sim.6927 26970107

[13] Uno H, Cai T, Pencina MJ (2011). On the C-statistics for evaluating overall adequacy of risk prediction procedures with censored survival data. Stat Med.

[14] Maxim LD, Niebo R, Utell MJ (2014). Screening tests: a review with examples. Inhal Toxicol.

[15] Liu RZ, Graham K, Glubrecht DD, Lai R, Mackey JR, Godbout R (2012). A fatty acid-binding protein 7/RXRbeta pathway enhances survival and proliferation in triple-negative breast cancer. J Pathol.

[16] Lee JH, Welch DR. Suppression of metastasis in human breast carcinoma MDA-MB-435 cells after transfection with the metastasis suppressor gene, KiSS-1. Cancer Res. 1997;57(12):2384–7. 9192814

[17] Zhang JY, Chan EK, Peng XX, Tan EM (1999). A novel cytoplasmic protein with RNA-binding motifs is an autoantigen in human hepatocellular carcinoma. J Exp Med.

[18] Le Moullec JM, Jullienne A, Chenais J, Lasmoles F, Guliana JM, Milhaud G, Moukhtar MS (1984). The complete sequence of human preprocalcitonin. FEBS Lett.

[19] Nagai Y, Aoki J, Sato T, Amano K, Matsuda Y, Arai H, Inoue K (1999). An alternative splicing form of phosphatidylserine-specific phospholipase A1 that exhibits lysophosphatidylserine-specific lysophospholipase activity in humans. J Biol Chem.

[20] Rasila T, Saavalainen O, Attalla H, Lankila P, Haglund C, Holtta E, Andersson LC (2019). Astroprincin (FAM171A1, C10orf38): a regulator of human cell shape and invasive growth. Am J Pathol.

[21] Wang Y, Li H, Ma J, Fang T, Li X, Liu J, Afewerky HK, Li X, Gao Q (2019). Integrated bioinformatics data analysis reveals prognostic significance of SIDT1 in triple-negative breast Cancer. Onco Targets Ther.

[22] Christgen M, Geffers R, Kreipe H, Lehmann U (2013). IPH-926 lobular breast cancer cells are triple-negative but their microarray profile uncovers a luminal subtype. Cancer Sci.

[23] Yi J, Ren L, Li D, Wu J, Li W, Du G, Wang J (2020). Trefoil factor 1 (TFF1) is a potential prognostic biomarker with functional significance in breast cancers. Biomed Pharmacother.

[24] Umesh A, Park J, Shima J, Delaney J, Wisotzkey R, Kelly E, Chiu EB, Madhusoodanan J, Shekar M, Kupershmidt I: Identification of AGR3 as a potential biomarker though public genomic data analysis of triple-negative (TN) versus triple-positive (TP) breast cancer (BC). Journal of Clinical Oncology Official Journal of the American Society of Clinical Oncology 2012, 30(27_suppl):31.

[25] Dai X, Ma R, Zhao X, Zhou F (2019). Epigenetic profiles capturing breast cancer stemness for triple negative breast cancer control. Epigenomics.

[26] Mukhopadhyay A, Khoury T, Stein L, Shrikant P, Sood AK (2013). Prostate derived Ets transcription factor and Carcinoembryonic antigen related cell adhesion molecule 6 constitute a highly active oncogenic axis in breast cancer. Oncotarget.

[27] Pan H, Peng Z, Lin J, Ren X, Zhang G, Cui Y (2018). Forkhead box C1 boosts triple-negative breast cancer metastasis through activating the transcription of chemokine receptor-4. Cancer Sci.

[28] Darbeheshti F, Rezaei N, Amoli MM, Mansoori Y, Tavakkoly Bazzaz J (2019). Integrative analyses of triple negative dysregulated transcripts compared with non-triple negative tumors and their functional and molecular interactions. J Cell Physiol.

[29] Watkins J, Weekes D, Shah V, Gazinska P, Joshi S, Sidhu B, Gillett C, Pinder S, Vanoli F, Jasin M (2015). Genomic complexity profiling reveals that HORMAD1 overexpression contributes to homologous recombination deficiency in triple-negative breast cancers. Cancer Discov.

[30] Yu KD, Zhu R, Zhan M, Rodriguez AA, Yang W, Wong S, Makris A, Lehmann BD, Chen X, Mayer I (2013). Identification of prognosis-relevant subgroups in patients with chemoresistant triple-negative breast cancer. Clin Cancer Res.

[31] Zhong G, Lou W, Shen Q, Yu K, Zheng Y (2020). Identification of key genes as potential biomarkers for triplenegative breast cancer using integrating genomics analysis. Mol Med Rep.

[32] Hammond ME, Hayes DF, Wolff AC, Mangu PB, Temin S (2010). American society of clinical oncology/college of american pathologists guideline recommendations for immunohistochemical testing of estrogen and progesterone receptors in breast cancer. J Oncol Pract.

[33] Wali VB, Patwardhan GA, Pelekanou V, Karn T, Cao J, Ocana A, Yan Q, Nelson B, Hatzis C, Pusztai L (2019). Identification and validation of a novel biologics target in triple negative breast Cancer. Sci Rep.

[34] Castilla M, López-García M, Atienza MR, Rosa-Rosa JM, Díaz-Martín J, Pecero ML, Vieites B, Romero-Pérez L, Benítez J, Calcabrini A (2014). VGLL1 expression is associated with a triple-negative basal-like phenotype in breast cancer. Endocr Relat Cancer.

[35] Segaert P, Lopes MB, Casimiro S, Vinga S, Rousseeuw PJ (2019). Robust identification of target genes and outliers in triple-negative breast cancer data. Stat Methods Med Res.

[36] Jinesh GG, Flores ER, Brohl AS (2018). Chromosome 19 miRNA cluster and CEBPB expression specifically mark and potentially drive triple negative breast cancers. PLoS One.

[37] Metcalf S, Dougherty S, Kruer T, Hasan N, Biyik-Sit R, Reynolds L, Clem BF. Selective loss of phosphoserine aminotransferase 1 (PSAT1) suppresses migration, invasion, and experimental metastasis in triple negative breast cancer. Clin Exp Metastasis. 2019. 10.1007/s10585-019-10000-7 31630284

[38] Coyle KM, Murphy JP, Vidovic D, Vaghar-Kashani A, Dean CA, Sultan M, Clements D, Wallace M, Thomas ML, Hundert A, et al. Breast cancer subtype dictates DNA methylation and ALDH1A3-mediated expression of tumor suppressor RARRES1. Oncotarget. 2016. 10.18632/oncotarget.9858 PMC5190082 27286452

[39] Kozin SV, Maimon N, Wang R, Gupta N, Munn L, Jain RK, Garkavtsev I (2017). Secretory leukocyte protease inhibitor (SLPI) as a potential target for inhibiting metastasis of triple-negative breast cancers. Oncotarget.

[40] Sizemore GM, Sizemore ST, Seachrist DD, Keri RA (2014). GABA(a) receptor pi (GABRP) stimulates basal-like breast Cancer cell migration through activation of extracellular-regulated kinase 1/2 (ERK1/2). J Biol Chem.

[41] Aushev V, Gopalakrishnan K, Teitelbaum SL, Parada H, Santella RM, Gammon M, Chen J. Tumor expression of environmental chemical-responsive genes and breast cancer mortality. Endocr Relat Cancer. 2019. 10.1530/ERC-19-0357 31593922

[42] Kelwick R, Wagstaff L, Decock J, Roghi C, Cooley LS, Robinson SD, Arnold H, Gavrilović J, Jaworski DM, Yamamoto K (2015). Metalloproteinase-dependent and -independent processes contribute to inhibition of breast cancer cell migration, angiogenesis and liver metastasis by a disintegrin and metalloproteinase with thrombospondin motifs-15. Int J Cancer.

[43] Cohen H, Ben-Hamo R, Gidoni M, Yitzhaki I, Kozol R, Zilberberg A, Efroni S (2014). Shift in GATA3 functions, and GATA3 mutations, control progression and clinical presentation in breast cancer. Breast Cancer Res.

[44] Scanlan MJ, Gout I, Gordon CM, Williamson B, Stockert E, Gure AO, Jäger D, Chen YT, Mackay A, O'Hare MJ et al. Humoral immunity to human breast cancer: antigen definition and quantitative analysis of mRNA expression. Cancer Immun. 2001;1:4. 12747765

[45] Stolk L, Zhai G, van Meurs JB, Verbiest MM, Visser JA, Estrada K, Rivadeneira F, Williams FM, Cherkas L, Deloukas P et al. Loci at chromosomes 13, 19 and 20 influence age at natural menopause. Nat Genet. 2009;41(6):645–7. 10.1038/ng.387 PMC3000545 19448619

[46] Zhang G, Chen M, Kai J, Ma Q, Zhong A, Xie S, Zheng H, Wang Y, Tong Y, Lu R, et al. Molecular profiling of mucinous epithelial ovarian cancer by weighted gene co-expression network analysis. Gene. 2019. 10.1016/j.gene.2019.05.034 31108164

[47] Shergalis A, Bankhead A, Luesakul U, Muangsin N, Neamati N (2018). Current challenges and opportunities in treating Glioblastoma. Pharmacol Rev.

[48] Naderi A. SRARP and HSPB7 are epigenetically-regulated gene pairs that function as tumor suppressors and predict clinical outcome in malignancies. Mol Oncol. 2018. 10.1002/1878-0261.12195 PMC5928383 29577611

[49] Atkinson A (1986). Masking unmasked. Biometrika.

[50] Nisbet R, Miner G, Yale K (2018). Handbook of statistical analysis and data mining applications.

[51] Kotu V, Deshpande B (2018). Data science concepts and practice.

[52] D'Agostino RB (1998). Propensity score methods for bias reduction in the comparison of a treatment to a non-randomized control group. Stat Med.

[53] MATLAB (2018). R2018a.

[54] R Core Team. R: A language and environment for statistical computing. 2019: URL https://www.R-project.org.

[55] Sevinc F, KURNAZ I, HOFFMANN, FILZMOSER P: enetLTS: Robust and Sparse Methods for High Dimensional Linear and Logistic Regression. *R package version 010* 2018: https://CRAN.R-project.org/package=enetLTS.

