# Supplementary material for: Comparison of methods for the detection of outliers and associated biomarkers in mislabeled omics data
Source: BMC Bioinformatics. 2020 Aug 14;21:357. doi: 10.1186/s12859-020-03653-9 (PMC7646480; doi:10.1186/s12859-020-03653-9)
Supplement: Supplementary file 1 — Additional file 1. [file 12859_2020_3653_MOESM1_ESM.docx]

Supplementary Material for “**Comparison of Methods for the Detection of Outliers and Associated Biomarkers in Mislabeled Omics Data”**

Hongwei Sun^1,2^, Yuehua Cui^3^ Hui Wang^1^ Haixia Liu^2^ and Tong Wang^1*†^

* Correspondence: tongwang@sxmu.edu.cn

^1^ Department of Health Statistics, School of Public Health, Shanxi Medical University, Taiyuan City, Shanxi 030001, China.

Index

[S1. Description of three methods 1](#_Toc40899242)

[S2. Simulation results for the comparison of the three methods 7](#_Toc40899243)

[S3. Results of Ensemble for the Analysis of TNBC dataset 10](#_Toc40899244)

[S4. Results of enetLTS for the Analysis of TNBC dataset 12](#_Toc40899245)

[S5. Results of Rlogreg for the Analysis of TNBC dataset 16](#_Toc40899246)

# S1. Description of three methods

Several models that deal with mislabeled samples are based on the logistic model, so the general model of logistic regression is provided first.

Let $\boldsymbol{y}={(y_{1},y_{2},\cdots,y_{n})}^{'}$ be the responses that follow the Bernoulli distribution independently, that is, $y_{i}\sim B(1,\pi_{i})$, where success probability $\pi_{i}$ depends on the value of *p*-dimensional covariate vector $\boldsymbol{x}_{\boldsymbol{i}}={(x_{i1},x_{i2},\cdots,x_{ip})}^{'}$. The logistic model is assumed to have the following form:

$P\left\{ y_{i}=1 | \boldsymbol{x}_{i} \right\}=F(\boldsymbol{x}_{\boldsymbol{i}}^{\boldsymbol{'}}\boldsymbol{\beta})=\frac{exp(\boldsymbol{x}_{\boldsymbol{i}}^{\boldsymbol{'}}\boldsymbol{\beta})}{1+exp(\boldsymbol{x}_{\boldsymbol{i}}^{\boldsymbol{'}}\boldsymbol{\beta})}$, (1)

where $\boldsymbol{\beta}=\left( \beta_{1},\beta_{2},\cdots,\beta_{p} \right)^{'}$denotes parameters of interest.

The negative log-likelihood of the logistic model is given by

$$\mathcal{l}\left( \boldsymbol{\beta} \right)=-\sum_{i=1}^{n} log\mathcal{(l(}y_{i},\boldsymbol{x}_{\boldsymbol{i}}^{\boldsymbol{'}}\boldsymbol{\beta))=}\sum_{i=1}^{n} d(y_{i},\boldsymbol{x}_{\boldsymbol{i}}^{\boldsymbol{'}}\boldsymbol{\beta)}$$

$\boldsymbol{=}\sum_{i=1}^{n} \{-y_{i}\boldsymbol{x}_{\boldsymbol{i}}^{\boldsymbol{'}}\boldsymbol{\beta+}log\boldsymbol{(}1\boldsymbol{+}exp(\boldsymbol{x}_{\boldsymbol{i}}^{\boldsymbol{'}}\boldsymbol{\beta))\},}$ (2)

where ${d(y_{i},\boldsymbol{x}}_{\boldsymbol{i}}^{\boldsymbol{'}}\boldsymbol{\beta)}$ is the deviance component. The maximum likelihood estimator ${\hat{\boldsymbol{\beta}}}^{ML}$ is defined as

${\hat{\boldsymbol{\beta}}}^{ML}={argmin}_{\boldsymbol{\beta}}\mathcal{l}\left( \boldsymbol{\beta} \right)={argmin}_{\boldsymbol{\beta}}\sum_{i=1}^{n} d{(y}_{i}\boldsymbol{,x}_{\boldsymbol{i}}^{\boldsymbol{'}}\boldsymbol{\beta}\boldsymbol{)}.$ (3)

**1 Sparse label-noise-robust logistic regression**

Bootkrajang et al. [[6](#_ENREF_6)] proposed Rlogreg to detect mislabeled samples, which is a robust extension of the sparse Blogreg.

**1.1 Label-noise robust logistic regression**

First, in a low-dimensional setting, Hansman et al. [[47](#_ENREF_47)] proposed a modified MLE that considers the label-flipping probabilities:

$$\gamma_{jk}=P\left( y_{i}=k | \tilde{y}_{i}=j \right),$$

which represents the probability that the label has flipped from true label *j* to observed label *k*, $\tilde{y}_{i}$ is for the true response of observation *i*. These parameters form a transition table, which we call the “gamma table” (denoted by $\Gamma$), and these label flipping probabilities can be estimated. Then for observed binary dependent variable $y_{i}$,

$$P\left( y_{i}=1 | x_{i} \right)=P(y_{i}=1|\tilde{y}_{i}=0)P\left( \tilde{y}_{i}=0 | x_{i} \right)+P(y_{i}=1|\tilde{y}_{i}=1)P\left( \tilde{y}_{i}=1 | x_{i} \right)$$

$$=\gamma_{01}(1-F(\boldsymbol{x}_{\boldsymbol{i}}^{\boldsymbol{T}}\boldsymbol{\beta}))+(1-\gamma_{10})F(\boldsymbol{x}_{\boldsymbol{i}}^{\boldsymbol{T}}\boldsymbol{\beta})$$

$${=\gamma}_{01}+(1-\gamma_{10}-\gamma_{01})F(\boldsymbol{x}_{\boldsymbol{i}}^{\boldsymbol{T}}\boldsymbol{\beta}).$$

Then the log-likelihood function of logistic regression with the mislabeled samples is

$\mathcal{l}\left( \boldsymbol{\beta,}\gamma_{01},\gamma_{10} \right)=\sum_{i=1}^{n} log\mathcal{(l(}y_{i},\boldsymbol{x}_{\boldsymbol{i}}^{\boldsymbol{T}}\boldsymbol{\beta}$**)**

$$\boldsymbol{=}\sum_{i=1}^{n} (y_{i}ln(\gamma_{01}+(1-\gamma_{01}-\gamma_{10})F(\boldsymbol{x}_{\boldsymbol{i}}^{\boldsymbol{T}}\boldsymbol{\beta}))$$

$\boldsymbol{+(}1-y_{i}\boldsymbol{)}ln\boldsymbol{(}1-\gamma_{01}+(1-\gamma_{01}-\gamma_{10})F(\boldsymbol{x}_{\boldsymbol{i}}^{\boldsymbol{T}}\boldsymbol{\beta})\boldsymbol{)),}$ **(5)**

which maximizes the log likelihood function $\mathcal{l}\left( \boldsymbol{\beta,}\gamma_{01},\gamma_{10} \right)$ derived the MLE of $(\boldsymbol{\beta,}\gamma_{01},\gamma_{10})$.

**1.2 Sparse label-noise-Robust Logistic Regression**

Bootkrajang et al. [[6](#_ENREF_6)] used an $L_{1}$ regularization term on the objective function to incorporate sparsity:

$\mathcal{l}\left( \boldsymbol{\beta,}\gamma_{01},\gamma_{10} \right)\boldsymbol{+}\lambda\boldsymbol{||\beta||}_{\boldsymbol{1}},$ **(6)**

where $\lambda$ is the regularization parameter that balances between fitting the data well and having small parameter values. $\lambda$ is typically chosen using cross-validation; however, in Bootkrajang et al. [[6](#_ENREF_6)], cross-validation was not used because of its computational burden, and it would be influenced by label noise. Bootkrajang et al. [[6](#_ENREF_6)] determined $\lambda$ by applying Jeffrey prior to $\lambda$, $p\left( \lambda\right)=1/\lambda$, and integrating it out of the model:

$$p\left( \boldsymbol{\beta} \right)=\int p\left( \boldsymbol{\beta}|\lambda\right)p\left( \lambda\right)d\lambda,$$

where $p\left( \boldsymbol{\beta}|\lambda\right)$ is the prior on $\boldsymbol{\beta,}$ which follows the Laplace distribution (see Cawley et al. [[48](#_ENREF_48)] for details). Finally, $\lambda$ is obtained

$\lambda=\frac{N}{\sum_{j=1}^{p} |\beta_{j}|}$, (7)

where *N* denotes the number of non-zero coefficients, that is, those with $\beta_{j}\neq0$.

Bootkrajang et al. [[6](#_ENREF_6)] used the Gauss–Seidel and coordinate-wise descent algorithm to optimize $\boldsymbol{\beta}$, and obtained label-flipping probabilities $\boldsymbol{\Gamma}$ by solving the stationary equation (see Bootkrajang et al. [[6](#_ENREF_6)]for details).

**1.3 Outlier detection**

Bootkrajang et al. [[6](#_ENREF_6)] introduced “a degree of belief” to detect outliers. In simple terms, the outliers detected by Rlogreg are the misclassified observations with response *y* = 1 that are predicted as zero, or ones with *y* = 0 but predicted as one. The predicted response is

$$\hat{y}_{i}=\left\{ \begin{matrix} 1 & if \hat{p}_{i}\geq0.5 \\ 0 & if\hat{p}_{i}<0.5 \end{matrix} \right.,$$

where predicted probability $\hat{p}_{i}=\frac{exp(\boldsymbol{x}_{i}^{'}\hat{\boldsymbol{\beta}})}{1+exp(\boldsymbol{x}_{i}^{'}\hat{\boldsymbol{\beta}})}$.

**2 Robust elastic net based on the least trimmed square**

Kurnaz et al. [[13](#_ENREF_13)] proposed enetLTS, which combines the LTS and EN penalty to obtain a robust estimation for linear and logistic regression. A reweighting step improves the efficiency of the estimator.

**2.1 Elastic net**

Zou and Hastie [[3](#_ENREF_3)] proposed EN, which combines both $L_{1}$ and $L_{2}$ penalties:

$${{\hat{\boldsymbol{\beta}}}^{PML}=argmin}_{\boldsymbol{\beta}}\sum_{i=1}^{n} d{(y}_{i}\boldsymbol{,x}_{\boldsymbol{i}}^{\boldsymbol{'}}\boldsymbol{\beta}\boldsymbol{)}+n\lambda P_{\alpha}(\boldsymbol{\beta})$$

$P_{\alpha}\left( \boldsymbol{\beta} \right)=\left( 1-\alpha\right)\frac{1}{2}{||\boldsymbol{\beta}||}_{2}+\alpha{||\boldsymbol{\beta}||}_{1}$ , (8)

Where $\lambda\geq0$ is a penalty parameter, and $\alpha$, which is the mixing proportion of the ridge and LASSO penalties, takes a value in [0, 1].The EN tends to select groups of correlated variables.

**2.2 Robust elastic net based on the least trimmed square**

However, likelihood-based procedures (8) are hugely sensitive to outliers, which causes biased estimation and inappropriate conclusions[[^13^](#_ENREF_13)].

Kurnaz et al. [[13](#_ENREF_13)] proposed enetLTS, which provides a robust estimation for EN-type penalized logistic regression through trimming and is defined as

${\hat{\boldsymbol{\beta}}}^{enetLTS}={argmin}_{\boldsymbol{\beta}}\sum_{l=1}^{h} d(y_{i_{l}}\boldsymbol{,x}_{i_{l}}^{\boldsymbol{'}}\boldsymbol{\beta}\boldsymbol{)}+h\lambda P_{\alpha}(\boldsymbol{\beta)}$, (9)

Where $d(y_{i_{1}}\boldsymbol{,x}_{i_{1}}^{\boldsymbol{'}}\boldsymbol{\beta}\boldsymbol{)\leq}d(y_{i_{2}}\boldsymbol{,x}_{i_{2}}^{\boldsymbol{'}}\boldsymbol{\beta}\boldsymbol{)\leq\cdots\leq}d{(y}_{i_{n}}\boldsymbol{,x}_{i_{n}}^{\boldsymbol{'}}\boldsymbol{\beta}\boldsymbol{)}$**,** $i_{l}\in\left\{ 1,2,\cdots,n \right\},$ where $d(y_{i_{k}}\boldsymbol{,x}_{i_{k}}^{\boldsymbol{'}}\boldsymbol{\beta}\boldsymbol{)}$ is the ordered deviance. *h* = [$\delta n$] ([.] means rounding down to the nearest integer.) and $\alpha\in\left[ 0.5,1 \right],$ where $1-\delta$ is the trimmed portion. Compared with EN, enetLTS only retains *h* observations with the smallest deviances, whereas *n-h* least likely observations under the given model are excluded.

The C-step algorithm has been used to identify this optimal subset, which includes *h* observations with the smallest deviances. It is based on the idea of repeatedly applying non-robust classical estimators to data subsets only.

**2.3 Reweighted step and outlier detection**

The LTS estimator is highly robust, but has low efficiency, and thus a weighting step is commonly used [[49](#_ENREF_49)]. In a reweighting step, according to the current model, the outliers are identified and downweighted.

Kurnaz et al. [[13](#_ENREF_13)] detected outliers using large Pearson residuals in the reweighted step. The Pearson residuals for logistic regression are approximately standard normally distributed and defined as

$$r_{i}=\frac{y_{i}-\hat{p}_{i}}{\sqrt{\hat{p}_{i}(1-\hat{p}_{i})}},$$

where $\hat{p}$ is the fitted value of $p_{i}$ and $y_{i}$ is the *i*th response.

We define a binary weight for the *i*th observation as

, (10)

where $\Phi(x)$ is the distribution function of a standard normal distribution. Kurnaz et al. [[13](#_ENREF_13)] set $\delta$=0.0125 and $\Phi^{-1}\left( 1-\delta\right)=2.24$, which means that observations with residuals beyond 2.24 are regarded as outliers. $H^{*}$is composed of observations that are not flagged as outliers.

The reweighted estimator $\hat{\beta}_{reweighted}$ is the solution of the EN-type penalized logistic regression performed on subset $H^{*}$, which is expressed as

${\hat{\boldsymbol{\beta}}}_{reweighted}\boldsymbol{=}arg\min_{\beta} \sum_{i=1}^{n} w_{i}d(y_{i},\boldsymbol{x}_{\boldsymbol{i}}^{\boldsymbol{'}}\boldsymbol{\beta}\mathbf{)}+{\lambda n}_{w}\sum_{j=1}^{p} P_{\alpha_{opt}}(\boldsymbol{\beta)},$ (11)

where$n_{w}=\sum_{i=1}^{n} w_{i}$. The updated parameter $\lambda$ is determined using 5-fold cross-validation, with the simplification that $\alpha_{opt}$ is already fixed.

**3 Ensemble**

The Ensemble strategy, which consists of three high-dimensional variable selection methods, that is, EN, SPLS-DA, and SGPLS, was proposed by Lopes et al. [[9](#_ENREF_9)]. The outlierness of observations are evaluated based on Cook’s D. Rank product statistics corrected for multiple testing is used to achieve consensus.

**3.1 Elastic net**

The first model in Ensemble is the EN, which is the same as Eq. (8).

**3.2 SPLS**

The second method in Ensemble is SPLS-DA and the third method is SGPLS, which are both based on PLS regression. In PLS regression, an orthogonal basis of latent variables – not directly observed or measured – is constructed in such a manner that they are maximally correlated with the response variable.[[5](#_ENREF_5)]

Within the course of PLS dimension reduction, Chung and Keles [[50](#_ENREF_50)] proposed sparse PLS (SPLS) regression, which promotes variable selection by solving the following minimization problem:

$\min_{w,c} \{-l\mathbf{w}^{T}\mathbf{Mw}+(1-l)(\mathbf{c}-\mathbf{w})^{T}\mathbf{M}(\mathbf{c}-\mathbf{w})+\lambda_{1}||\mathbf{c}||_{1}+\lambda_{2}||\mathbf{c}||_{2}\}$ , (12)

subject to $\mathbf{w}^{T}\mathbf{w}=1$, where $\mathbf{M}=\mathbf{X}^{T}\mathbf{YY}^{T}\mathbf{X}$. This formulation promotes

the exact zero property by imposing the $L_{1}$ penalty onto a surrogate of the direction vector ($\mathbf{c}$) instead of the original direction vector ($\mathbf{w}$), while keeping $\mathbf{w}$ and $\mathbf{c}$close to each other. The $L_{2}$ penalty eliminates the potential singularity of$\mathbf{M}$.

**3.3 SPLS-DA**

PLS can also be applied to the dataset whose response variable is categorical. Chung and Keles [21] proposed two methods to extend SPLS to classification. The first is the SPLS-DA, which is a two-stage procedure. In the first stage, SPLS regression is used to construct latent variables by considering the categorical response as a continuous variable; for a binary response, a dummy {0,1} code is used. In the second stage, because the number of latent variables *L* is typically much smaller than sample size *n*, a linear classifier, such as linear discriminant analysis or logistic regression, is applied.

**3.4 SGPLS**

SGPLS extends SPLS to the generalized linear model (GLM) framework. The minimization problem in Eq.(2) can be solved using the Newton–Raphson algorithm, which results in iteratively reweighted least squares [[50](#_ENREF_50)]. SPLS can be incorporated into the GLM framework by solving this weighted least squares problem:

$\min_{\beta} \sum_{i=1}^{n} \nu_{i}(z_{i}-\mathbf{x}_{i}^{T}\mathbf{\beta})^{2}$, (13)

where$v_{i}=p_{i}(1-p_{i})$ and$z_{i}=x_{i}^{T}\beta+(y_{i}-p_{i})/v_{i}$β (the working response). The direction vectors of SGPLS are obtained by solving Eq. (12) subject to$w^{T}w=1$, where$\mathbf{M}=\mathbf{X}^{T}\mathbf{Vzz}^{T}\mathbf{V}^{T}\mathbf{X}$, where $\mathbf{V}$ is the diagonal matrix with entries$v_{i}$ and $\mathbf{z}=(z_{1},\cdots,z_{n})$ is the vector of working responses.

**3.5 Ensemble outlier detection using the rank product test**

Cook’s D [[51](#_ENREF_51), [52](#_ENREF_52)] is a measure of influence that is widely used in outlier detection that combines information about the leverage (a measure of how far a predictor deviates from its mean) and residual (the difference between the predicted and actual values of response). After outlierness rankings based on Cook’s D is obtained from various modeling strategies, the rank product test is used to obtain a consensus ranking of observations, $RP_{i}=\prod_{j=1}^{k} R_{ij}$,$R_{ij}=rank(D_{ij})$, where$D_{ij}$is the Cook’s D of the $i^{th}$ observation (i = 1, . . . , n) obtained using the $j^{th}$ model[[53](#_ENREF_53)]. The q-value is used to control the false discovery rate (*FDR*) of outlier detection, which is a correction method that deals with the multiple testing problem [[54](#_ENREF_54)] .

# S2. Simulation results for the comparison of the three methods

**Table** S2-1 Results of the three methods for the datasets with *n*=100 and *p*=200.

| $\boldsymbol{\varepsilon}$ | *Methods* | Variables selection | | | |  | Outliers detection | | |  | Prediction |
| --- | --- | --- | --- | --- | --- | --- | --- | --- | --- | --- | --- |
|  |  | *Model size* | *PSR* | *FDR* | *GM* |  | *Num* | *Sn* | *FPR* |  | *MR* |
|  | Rlogreg | 14.64 | 0.292 | 0.402 | 0.417 |  | 2.77 | 0.65 | 0.015 |  | 0.104 |
| 0.02 | enetLTS | 55.05 | 0.695 | 0.530 | 0.546 |  | 2.75 | 0.61 | 0.016 |  | 0.106 |
|  | Ensemble | 11.07 | 0.364 | 0.169 | 0.540 |  | 1.40 | 0.49 | 0.004 |  | - |
|  | Rlogreg | 14.49 | 0.478 | 0.253 | 0.363 |  | 4.22 | 0.615 | 0.018 |  | 0.122 |
| 0.05 | enetLTS | 59.02 | 0.698 | 0.548 | 0.534 |  | 4.12 | 0.600 | 0.018 |  | 0.115 |
|  | Ensemble | 13.34 | 0.431 | 0.024 | 0.631 |  | 2.45 | 0.507 | 0.004 |  | - |
|  | Rlogreg | 13.96 | 0.190 | 0.587 | 0.279 |  | 21.65 | 0.839 | 0.155 |  | 0.154 |
| 0.1 | enetLTS | 71.75 | 0.704 | 0.623 | 0.483 |  | 7.13 | 0.529 | 0.026 |  | 0.141 |
|  | Ensemble | 11.00 | 0.349 | 0.042 | 0.555 |  | 3.47 | 0.353 | 0.003 |  | - |
|  | Rlogreg | 13.05 | 0.158 | 0.637 | 0.239 |  | 10.85 | 0.517 | 0.036 |  | 0.179 |
| 0.15 | enetLTS | 64.6 | 0.650 | 0.624 | 0.472 |  | 9.80 | 0.473 | 0.032 |  | 0.170 |
|  | Ensemble | 6.29 | 0.206 | 0.132 | 0.404 |  | 3.53 | 0.227 | 0.001 |  | - |

**Notes:**$\varepsilon$, the proportion of outliers.

**Abbreviations**: *PSR*, Positive Selection Rate. *FDR*, False Discovery Rate. *Sn*, sensitivity. *FPR*, False Positive Rate. *MR*, Misclassification Rate. *Num*, number of outliers. *GM*, the geometric mean of *PSR* and (1-*FDR*).

**Table** S2-2 Results of the three methods for the datasets with *n*=100 and *p*=1000.

| $\boldsymbol{\varepsilon}$ | *Methods* | Variables selection | | | |  | Outliers detection | | |  | Prediction |
| --- | --- | --- | --- | --- | --- | --- | --- | --- | --- | --- | --- |
|  |  | *Model size* | *PSR* | *FDR* | *GM* |  | *Num* | *Sn* | *FPR* |  | *MR* |
|  | Rlogreg | 17.72 | 0.237 | 0.597 | 0.309 |  | 1.41 | 0.445 | 0.005 |  | 0.122 |
| 0.02 | enetLTS | 68.55 | 0.586 | 0.636 | 0.442 |  | 2.10 | 0.500 | 0.011 |  | 0.118 |
|  | Ensemble | 14.08 | 0.457 | 0.020 | 0.649 |  | 1.64 | 0.594 | 0.005 |  | - |
|  | Rlogreg | 17.97 | 0.200 | 0.671 | 0.256 |  | 3.08 | 0.405 | 0.016 |  | 0.148 |
| 0.05 | enetLTS | 92.99 | 0.636 | 0.683 | 0.422 |  | 3.33 | 0.468 | 0.015 |  | 0.134 |
|  | Ensemble | 14.91 | 0.491 | 0.022 | 0.673 |  | 2.38 | 0.520 | 0.003 |  | - |
|  | Rlogreg | 18.21 | 0.131 | 0.782 | 0.206 |  | 3.01 | 0.230 | 0.010 |  | 0.169 |
| 0.1 | enetLTS | 105.03 | 0.585 | 0.718 | 0.369 |  | 5.42 | 0.407 | 0.019 |  | 0.163 |
|  | Ensemble | 11.26 | 0.355 | 0.052 | 0.562 |  | 3.63 | 0.393 | 0.001 |  | - |
|  | Rlogreg | 17.7 | 0.087 | 0.852 | 0.113 |  | 4.65 | 0.213 | 0.017 |  | 0.287 |
| 0.15 | enetLTS | 139.1 | 0.617 | 0.770 | 0.384 |  | 7.80 | 0.373 | 0.026 |  | 0.203 |
|  | Ensemble | 12.18 | 0.375 | 0.054 | 0.573 |  | 3.765 | 0.247 | 0.001 |  | - |

**Notes:**$\varepsilon$, the proportion of outliers.

**Abbreviations**: *PSR*, Positive Selection Rate. *FDR*, False Discovery Rate. *Sn*, sensitivity. *FPR*, False Positive Rate. *MR*, Misclassification Rate. *Num*, number of outliers. *GM*, the geometric mean of *PSR* and (1-*FDR*).

**Table** S2-3 Results of the three methods for the datasets with *n*=500 and *p*=200.

| $\boldsymbol{\varepsilon}$ | *Methods* | Variables selection | | | |  | Outliers detection | | |  | Prediction |
| --- | --- | --- | --- | --- | --- | --- | --- | --- | --- | --- | --- |
|  |  | *Model size* | *PSR* | *FDR* | *GM* |  | *Num* | *Sn* | *FPR* |  | *MR* |
|  | Rlogreg | 18.77 | 0.307 | 0.504 | 0.389 |  | 13.14 | 0.79 | 0.019 |  | 0.068 |
| 0.01 | enetLTS | 49.71 | 0.831 | 0.478 | 0.651 |  | 7.21 | 0.79 | 0.007 |  | 0.052 |
|  | Ensemble | 24.9 | 0.821 | 0.010 | 0.899 |  | 5.56 | 0.70 | 0.004 |  | - |
|  | Rlogreg | 18.28 | 0.288 | 0.519 | 0.371 |  | 18.40 | 0.806 | 0.021 |  | 0.074 |
| 0.02 | enetLTS | 45.89 | 0.798 | 0.460 | 0.653 |  | 12.41 | 0.800 | 0.009 |  | 0.056 |
|  | Ensemble | 24.0 | 0.790 | 0.011 | 0.880 |  | 7.80 | 0.651 | 0.003 |  | - |
|  | Rlogreg | 18.61 | 0.275 | 0.547 | 0.351 |  | 31.08 | 0.808 | 0.025 |  | 0.080 |
| 0.05 | enetLTS | 54.70 | 0.787 | 0.526 | 0.602 |  | 25.44 | 0.769 | 0.015 |  | 0.068 |
|  | Ensemble | 0.489 | 0.673 | 0.013 | 0.811 |  | 12.59 | 0.507 | 0.001 |  | - |
|  | Rlogreg | 19.63 | 0.261 | 0.593 | 0.324 |  | 53.06 | 0.793 | 0.031 |  | 0.089 |
| 0.1 | enetLTS | 65.03 | 0.774 | 0.548 | 0.548 |  | 47.49 | 0.759 | 0.023 |  | 0.086 |
|  | Ensemble | 17.27 | 0.568 | 0.013 | 0.741 |  | 16.76 | 0.339 | 0.0001 |  | - |
|  | Rlogreg | 18.28 | 0.288 | 0.519 | 0.371 |  | 18.4 | 0.806 | 0.021 |  | 0.074 |
| 0.15 | enetLTS | 60.67 | 0.737 | 0.598 | 0.534 |  | 69.78 | 0.753 | 0.031 |  | 0.101 |
|  | Ensemble | 15.24 | 0.504 | 0.005 | 0.698 |  | 19.76 | 0.263 | 0.0001 |  |  |

**Notes:**$\varepsilon$, the proportion of outliers.

**Abbreviations**: *PSR*, Positive Selection Rate. *FDR*, False Discovery Rate. *Sn*, sensitivity. *FPR*, False Positive Rate. *MR*, Misclassification Rate. *Num*, number of outliers. *GM*, the geometric mean of *PSR* and (1-*FDR*).

**Table** S2-4 Results of the three methods for the datasets with *n*=500 and *p*=1,000.

| $\boldsymbol{\varepsilon}$ | *Methods* | Variables selection | | | |  | Outliers detection | | |  | | Prediction |
| --- | --- | --- | --- | --- | --- | --- | --- | --- | --- | --- | --- | --- |
|  |  | *Model size* | *PSR* | *FDR* | *GM* |  | *Num* | *Sn* | *FPR* | |  | *MR* |
|  | Rlogreg | 16.32 | 0.199 | 0.629 | 0.271 |  | 12.76 | 0.662 | 0.019 | |  | 0.095 |
| 0.01 | enetLTS | 69.05 | 0.772 | 0.647 | 0.517 |  | 6.35 | 0.720 | 0.006 | |  | 0.0611 |
|  | Ensemble | 23.93 | 0.791 | 0.008 | 0.883 |  | 5.37 | 0.684 | 0.004 | |  | - |
|  | Rlogreg | 16.21 | 0.194 | 0.633 | 0.265 |  | 18.14 | 0.701 | 0.023 | |  | 0.100 |
| 0.02 | enetLTS | 69.90 | 0.730 | 0.679 | 0.482 |  | 12.25 | 0.780 | 0.009 | |  | 0.067 |
|  | Ensemble | 23.56 | 0.777 | 0.010 | 0.873 |  | 7.71 | 0.651 | 0.002 | |  | - |
|  | Rlogreg | 15.97 | 0.179 | 0.650 | 0.249 |  | 30.04 | 0.705 | 0.028 | |  | 0.110 |
| 0.05 | enetLTS | 68.4 | 0.719 | 0.669 | 0.483 |  | 20.88 | 0.737 | 0.007 | |  | 0.068 |
|  | Ensemble | 21.46 | 0.710 | 0.007 | 0.835 |  | 12.64 | 0.508 | 0.001 | |  | - |
|  | Rlogreg | 16.24 | 0.161 | 0.690 | 0.222 |  | 50.08 | 0.693 | 0.036 | |  | 0.124 |
| 0.1 | enetLTS | 89.77 | 0.695 | 0.749 | 0.411 |  | 40.31 | 0.674 | 0.016 | |  | 0.089 |
|  | Ensemble | 18.42 | 0.610 | 0.008 | 0.771 |  | 33.68 | 0.393 | 0.0003 | |  | - |
|  | Rlogreg | 17.75 | 0.145 | 0.734 | 0.195 |  | 69.15 | 0.657 | 0.047 | |  | 0.153 |
| 0.15 | enetLTS | 135.78 | 0.670 | 0.843 | 0.320 |  | 56.33 | 0.584 | 0.030 | |  | 0.137 |
|  | Ensemble | 16.06 | 0.533 | 0.003 | 0.714 |  | 19.89 | 0.263 | 0.0003 | |  | - |

**Notes:**$\varepsilon$, the proportion of outliers.

**Abbreviations**: *PSR*, Positive Selection Rate. *FDR*, False Discovery Rate. *Sn*, sensitivity. *FPR*, False Positive Rate. *MR*, Misclassification Rate. *Num*, number of outliers. *GM*, the geometric mean of *PSR* and (1-*FDR*).

**Table** S2-5 Outlier detection accuracy of the three methods for the simulated datasets based on the TNBC dataset (*n*=1000, *p*=5000).

| $\boldsymbol{\varepsilon}$ | *Methods* |  | Outliers detection | | |  | |
| --- | --- | --- | --- | --- | --- | --- | --- |
|  |  |  | *Num* | *Sn* | *FPR* | |  |
|  | Rlogreg |  | 97.6 | 0.868 | 0.079 | |  |
| 0.025 | enetLTS |  | 29.4 | 0.900 | 0.007 | |  |
|  | Ensemble |  | 20.8 | 0.732 | 0.003 | |  |
|  | Rlogreg |  | 119.3 | 0.856 | 0.081 | |  |
| 0.05 | enetLTS |  | 50.6 | 0.902 | 0.006 | |  |
|  | Ensemble |  | 27.7 | 0.536 | 0.001 | |  |
|  | Rlogreg |  | 158.6 | 0.843 | 0.083 | |  |
| 0.1 | enetLTS |  | 95.3 | 0.852 | 0.007 | |  |
|  | Ensemble |  | 29.7 | 0.295 | 0.0002 | |  |
|  | Rlogreg |  | 192.6 | 0.805 | 0.084 | |  |
| 0.15 | enetLTS |  | 139.0 | 0.889 | 0.007 | |  |
|  | Ensemble |  | 38.9 | 0.258 | 0.0002 | |  |

**Notes:**$\varepsilon$*,*the proportion of outliers.

**Abbreviations**: *Sn*, sensitivity. *FPR*, False Positive Rate. *Num*, number of outliers.

**Table S2-6** Results of the three methods for the datasets with leverage samples (*n*=500, *p*=1,000, and $\boldsymbol{\varepsilon}$=0.02 and $\boldsymbol{\varepsilon}$=0.05).

| $\boldsymbol{\varepsilon}$ | *Methods* | | Variables selection | | | |  | Outliers detection | | |  | | Prediction |
| --- | --- | --- | --- | --- | --- | --- | --- | --- | --- | --- | --- | --- | --- |
|  |  |  | *Model size* | *PSR* | *FDR* | *GM* |  | *Num* | *Sn* | *FPR* | |  | *MR* |
|  | Rlogreg | 14.0 | | 0.201 | 0.564 | 0.295 |  | 21.68 | 0.700 | 0.030 | |  | 0.087 |
| 0.02 | enetLTS | 65.3 | | 0.707 | 0.644 | 0.495 |  | 9.27 | 0.627 | 0.006 | |  | 0.061 |
|  | Ensemble | 22.4 | | 0.743 | 0.006 | 0.846 |  | 6.63 | 0.545 | 0.002 | |  | - |
|  | Rlogreg | 12.41 | | 0.184 | 0.548 | 0.288 |  | 53.52 | 0.667 | 0.079 | |  | 0.094 |
| 0.05 | enetLTS | 103.0 | | 0.733 | 0.773 | 0443. |  | 19.6 | 0.337 | 0.016 | |  | 0.086 |
|  | Ensemble | 26.5 | | 0.708 | 0.072 | 0.798 |  | 6.37 | 0.248 | 0.001 | |  | - |

# S3. Results of Ensemble for the Analysis of TNBC dataset

**Table** S3-1 Thirty outliers detected using Ensemble for the TNBC dataset, including the expression values, IHC, and FISH tests of ER, PR, and HER2(individuals highlighted in bold are suspect individuals.)

| **ID** | **ESR1** | **PGR** | **HER2** | **HER2_level** | **HER2_status** | **HER2_FISH** | **y** | **qvalues** |
| --- | --- | --- | --- | --- | --- | --- | --- | --- |
| TCGA-E9-A1ND | 1.44 (-) | 0.05 (-) | 13.05 |  | + |  | non-TNBC | 8.90E-06 |
| TCGA-AR-A1AJ | 1.47 (+) | 0.07 (-) | 9.74 |  | - |  | non-TNBC | 8.46E-06 |
| **TCGA-A2-A04U** | **0.02 (-)** | **0.02 (-)** | **9.64** | **1+** | **-** | **+** | **non-TNBC** | **1.65E-05** |
| TCGA-E9-A22G | 0.44 (-) | 0.02 (-) | 15.32 |  | + |  | non-TNBC | 2.76E-05 |
| TCGA-OL-A97C | 16.25 (-) | 8.56 (-) | 24.04 |  |  | - | TNBC | 7.43E-05 |
| TCGA-AC-A62X | 0.19 (+) | 0.02 (-) | 28.53 |  |  |  | non-TNBC | 4.24E-05 |
| TCGA-A7-A13D | 0.52 (-) | 0.81 (+) | 42.28 | 2+ | Equiv | - | non-TNBC | 6.89E-05 |
| TCGA-BH-A42U | 9.19 (-) | 1.83 (-) | 38.37 |  | - |  | TNBC | 7.16E-05 |
| TCGA-OL-A5S0 | 0.09 (+) | 0.06 (-) | 31.92 |  |  | + | non-TNBC | 7.07E-05 |
| TCGA-E2-A1II | 0.14 (-) | 0.19 (+) | 10.73 | 1+ | - |  | non-TNBC | 1.50E-04 |
| **TCGA-A2-A0EQ** | **2.13 (-)** | **0.04 (-)** | **30.15** | **3+** | **+** | **-** | **TNBC** | **1.62E-04** |
| TCGA-B6-A0IJ | 1.18 (+) | 0.46 (+) | 11.12 |  |  |  | non-TNBC | 1.55E-04 |
| TCGA-C8-A26Y | 0.12 (-) | 0.05 (-) | 22.92 | 1+ | - |  | TNBC | 1.53E-04 |
| TCGA-A2-A1G6 | 23.90 (-) | 21.45 (-) | 29.74 | 1+ | - |  | TNBC | 2.24E-04 |
| TCGA-BH-A5IZ | 5.12 (+) | 0.03 (-) | 28.08 |  | - | - | non-TNBC | 2.18E-04 |
| TCGA-A2-A0YJ | 0.09 (+) | 0.03 (-) | 240.24 | 0 | - |  | non-TNBC | 3.42E-04 |
| TCGA-AR-A1AH | 0.03 (+) | 0.03 (-) | 34.12 |  | - |  | non-TNBC | 2.68E-04 |
| TCGA-BH-A0DL | 6.99 (+) | 0.04 (-) | 9.92 |  | - |  | non-TNBC | 3.23E-04 |
| TCGA-E9-A1NC | 0.11 (-) | 0.07 (+) | 15.91 |  | + |  | non-TNBC | 2.76E-04 |
| TCGA-AO-A03U | 0.56 (-) | 0.12 (-) | 17.06 | 0 | - | - | TNBC | 2.97E-04 |
| TCGA-S3-AA0Z | 16.67 (+) | 0.07 (+) | 33.07 | 1+ | Equiv | - | non-TNBC | 3.42E-04 |
| TCGA-C8-A3M7 | 4.27 (-) | 0.76 (-) | 25.47 |  | - |  | TNBC | 3.14E-04 |
| TCGA-A7-A13E | 0.82 (+) | 0.06 (-) | 46.08 | 2+ | Equiv | - | non-TNBC | 3.88E-04 |
| TCGA-A2-A3Y0 | 2.18 (+) | 0.03 (-) | 11.34 | 1+ | - |  | non-TNBC | 5.12E-04 |
| TCGA-EW-A1OV | 0.23 (-) | 0.03 (-) | 28.91 |  | - | - | TNBC | 5.58E-04 |
| **TCGA-LL-A5YP** | **0.16 (+)** | **0.05 (-)** | **15.10** | **1+** | **-** | **+** | **non-TNBC** | **6.12E-04** |
| TCGA-LL-A6FR | 0.33 (-) | 0.04 (+) | 32.13 | 2+ | Equiv | + | non-TNBC | 9.14E-04 |
| TCGA-BH-A1EW | 29.98 (-) | 18.90 (-) | 42.47 |  | - |  | TNBC | 1.13E-03 |
| TCGA-D8-A1JF | 1.26 (-) | 0.11 (-) | 32.93 | 1+ | - |  | TNBC | 1.52E-03 |
| TCGA-A2-A1G1 | 0.53 (-) | 0.17 (-) | 819.76 | 2+ | Equiv | + | non-TNBC | 1.59E-03 |

T**able** S3-2 Genes selected by Ensemble for the TNBC dataset.

| Selected by three methods^*^ | CA12, ESR1, AGR2, TFF1, AGR3 |
| --- | --- |
| Selected by two methods^**^ | GATA3, MLPH, SLC44A4, GABRP, VGLL1, SCGB2A2, SCGB1D2, FOXA1, TFF3, FDCSP, ASPN, SLPI, MMP7, ERBB2, PGAP3, KLK6 |

*:Intersect of variables selected by three methods in Ensemble, i.e., EN, SPLS-DA, SGPLS. **: Intersect of variables selected by any two methods in Ensemble, i.e., EN, SPLS-DA, SGPLS.

**Table** S3-3 The genes and outliers in common with Lopes M B, et al. ^[1]^

| 6 genes | CA12, ESR1, ASPN, SLPI, PGAP3, KLK6 |
| --- | --- |
| 18 outliers | TCGA-E9-A1ND, TCGA-AR-A1AJ, TCGA-E9-A22G, TCGA-OL-A97C, TCGA-AC-A62X, TCGA-BH-A42U, TCGA-OL-A5S0, TCGA-E2-A1II, TCGA-A2-A0EQ, TCGA-A2-A1G6, TCGA-A2-A0YJ, TCGA-AR-A1AH, TCGA-C8-A3M7, TCGA-A2-A3Y0, TCGA-LL-A5YP, TCGA-D8-A1JF |

**Table** S3-4 Intersection of outliers detected by Ensemble, RLogreg and enetLTS.

| Intersection of three methods | TCGA-OL-A5S0, TCGA-E9-A22G, TCGA-AC-A62X, TCGA-A7-A13E, TCGA-E9-A1NC, TCGA-LL-A5YP, TCGA-A2-A0YJ, TCGA-A2-A1G1, TCGA-A2-A04U, TCGA-LL-A6FR, TCGA-B6-A0IJ, TCGA-BH-A5IZ, TCGA-AR-A1AJ, TCGA-E2-A1II, TCGA-AR-A1AH, TCGA-E9-A1ND, TCGA-S3-AA0Z, TCGA-A7-A13D, TCGA-A2-A3Y0 |
| --- | --- |
| Intersection of Ensemble and enetLTS except for the ones above | TCGA-BH-A0DL, TCGA-C8-A3M7, TCGA-BH-A1EW |

**Table** S3-5 Thirty-three Outliers detected by Ensemble for analysis for dataset with 5000 variables selected randomly(individuals highlighted in bold are suspect individuals.)

| TCGA-E2-A1II, TCGA-A7-A5ZV, TCGA-B6-A0IJ, TCGA-E9-A1ND, TCGA-BH-A42U, TCGA-AR-A1AJ, **TCGA-A2-A0EQ**, TCGA-A2-A3Y0, TCGA-A2-A1G6, TCGA-EW-A1OV, TCGA-D8-A1JK, TCGA-C8-A26Y, TCGA-D8-A1JF, TCGA-OL-A5S0, TCGA-BH-A5IZ, TCGA-E9-A22G, TCGA-AC-A62X, TCGA-AR-A1AH, TCGA-E9-A1NC, **TCGA-LL-A5YP**, TCGA-C8-A3M7, TCGA-OL-A97C, TCGA-E2-A1LG, TCGA-AC-A2QJ, TCGA-A2-A0YJ, TCGA-BH-A1EW, **TCGA-AN-A0FJ**, **TCGA-A2-A04U**, TCGA-A2-A3XV, TCGA-AR-A251, TCGA-E2-A1L7, TCGA-BH-A0DL, TCGA-S3-AA0Z |
| --- |

Table S3-6 Intersection of outliers detected by Ensemble for dataset with random variables and for the original TNBC dataset.

| 25 outliers | TCGA-E9-A1ND, TCGA-AR-A1AJ, TCGA-A2-A04U, TCGA-E9-A22G, TCGA-OL-A97C, TCGA-AC-A62X, TCGA-BH-A42U, TCGA-OL-A5S0, TCGA-E2-A1II, TCGA-A2-A0EQ, TCGA-B6-A0IJ, TCGA-C8-A26Y, TCGA-A2-A1G6, TCGA-BH-A5IZ, TCGA-A2-A0YJ, TCGA-AR-A1AH, TCGA-BH-A0DL, TCGA-E9-A1NC, TCGA-S3-AA0Z, TCGA-C8-A3M7, TCGA-A2-A3Y0, TCGA-EW-A1OV, TCGA-LL-A5YP, TCGA-BH-A1EW, TCGA-D8-A1JF |
| --- | --- |

Table S3-7 Gene identified by Ensemble for analysis for dataset with 5000 variables selected randomly(individuals highlighted in bold are in common with the original TNBC dataset.)

| [ELF5](https://www.genenames.org/data/gene-symbol-report/#!/hgnc_id/HGNC:3320), [DEFB1](https://www.genenames.org/data/gene-symbol-report/#!/hgnc_id/HGNC:2766), [PPP1R14C](https://www.genenames.org/data/gene-symbol-report/#!/hgnc_id/HGNC:14952), [MMP7](https://www.genenames.org/data/gene-symbol-report/#!/hgnc_id/HGNC:7174), [**TFF1**](https://www.genenames.org/data/gene-symbol-report/#!/hgnc_id/HGNC:11755), [TMSB15A](https://www.genenames.org/data/gene-symbol-report/#!/hgnc_id/HGNC:30744), [**AGR2**](https://www.genenames.org/data/gene-symbol-report/#!/hgnc_id/HGNC:328), [COL9A3](https://www.genenames.org/data/gene-symbol-report/#!/hgnc_id/HGNC:2219), [ERBB2](https://www.genenames.org/data/gene-symbol-report/#!/hgnc_id/HGNC:3430), [OLFM4](https://www.genenames.org/data/gene-symbol-report/#!/hgnc_id/HGNC:17190) |
| --- |

# S4. Results of enetLTS for the Analysis of TNBC dataset

**Table S**4-1 Sixty-eight outliers detected using enetLTS for the TNBC dataset, including the expression values, IHC, and FISH tests of ER, PR, and HER2(individuals highlighted in bold are suspect individuals.)

| ID | ESR1 | PGR | HER2 | HER2_level | HER2_status | HER2_FISH | y | Perres^*^ |
| --- | --- | --- | --- | --- | --- | --- | --- | --- |
| TCGA-E9-A22G | 0.44(-) | 0.02 (-) | 15.32 |  | + |  | non-TNBC | 37.22 |
| TCGA-A2-A0YJ | 0.09(+) | 0.03 (-) | 240.24 | 0 | - |  | non-TNBC | 35.66 |
| TCGA-A7-A13E | 0.82(+) | 0.06 (-) | 46.08 | 2+ | Equiv | - | non-TNBC | 33.23 |
| **TCGA-A2-A04U** | **0.02(-)** | **0.02 (-)** | **9.64** | **1+** | **-** | **+** | **non-TNBC** | **32.24** |
| TCGA-AR-A0TP | 0.04(+) | 0.03 (-) | 13.39 |  | - |  | non-TNBC | 32.15 |
| **TCGA-AN-A0FX** | **1.13(-)** | **0.64 (-)** | **24.02** | **1+** | **+** |  | **non-TNBC** | **30.70** |
| TCGA-AR-A251 | 1.57(+) | 0.10 (-) | 14.02 | 2+ | Equiv | - | non-TNBC | 30.23 |
| TCGA-LL-A6FR | 0.33(-) | 0.04 (+) | 32.13 | 2+ | Equiv | + | non-TNBC | 30.12 |
| TCGA-AC-A62X | 0.19(+) | 0.02 (-) | 28.53 |  |  |  | **non-TNBC** | 30.11 |
| TCGA-BH-A5IZ | 5.12(+) | 0.03 (-) | 28.08 |  | - | - | non-TNBC | 29.99 |
| TCGA-OL-A5S0 | 0.09(+) | 0.06 (-) | 31.92 |  |  | + | non-TNBC | 29.50 |
| **TCGA-LL-A5YP** | **0.16(+)** | **0.05 (-)** | **15.10** | **1+** | **-** | **+** | **non-TNBC** | **29.05** |
| TCGA-LL-A8F5 | 1.08(+) | 0.04 (-) | 11.86 | 1+ | - |  | non-TNBC | 27.77 |
| TCGA-B6-A0IJ | 1.18(+) | 0.46 (+) | 11.12 |  |  |  | non-TNBC | 27.74 |
| TCGA-A7-A13D | 0.52(-) | 0.81 (+) | 42.28 | 2+ | Equiv | - | non-TNBC | 27.59 |
| TCGA-AR-A24Q | 1.00(+) | 0.36 (-) | 20.67 |  | - |  | non-TNBC | 27.44 |
| TCGA-S3-AA0Z | 16.67(+) | 0.07 (+) | 33.07 | 1+ | Equiv | - | non-TNBC | 27.31 |
| **TCGA-AN-A0FJ** | **0.08(+)** | **0.04 (-)** | **14.28** | **1+** | **+** |  | **non-TNBC** | **27.29** |
| TCGA-AR-A1AH | 0.03(+) | 0.03 (-) | 34.12 |  | - |  | non-TNBC | 27.04 |
| TCGA-D8-A1JM | 5.00(+) | 0.01 (-) | 21.85 | 1+ | - |  | non-TNBC | 26.93 |
| TCGA-E9-A1NC | 0.11(-) | 0.07 (+) | 15.91 |  | + |  | non-TNBC | 26.84 |
| TCGA-A2-A3Y0 | 2.18(+) | 0.03 (-) | 11.34 | 1+ | - |  | non-TNBC | 26.35 |
| TCGA-D8-A1XW | 0.32(-) | 0.11 (+) | 21.03 | 1+ | - |  | non-TNBC | 24.28 |
| TCGA-AR-A1AJ | 1.47(+) | 0.07 (-) | 9.74 |  | - |  | non-TNBC | 24.07 |
| TCGA-A2-A1G1 | 0.53(-) | 0.17 (-) | 819.76 | 2+ | Equiv | + | non-TNBC | 23.70 |
| **TCGA-AO-A0JL** | **0.63(-)** | **0.08 (-)** | **63.60** | **1+** | **-** | **+** | **non-TNBC** | **23.68** |
| TCGA-A2-A4RX | 0.68(+) | 0.93 (+) | 26.64 | 1+ | - |  | non-TNBC | 23.43 |
| TCGA-E2-A14Y | 0.67(+) | 0.03 (+) | 487.90 | 2+ | Equiv | + | non-TNBC | 22.55 |
| TCGA-E2-A1II | 0.14(-) | 0.19 (+) | 10.73 | 1+ | - |  | non-TNBC | 22.18 |
| **TCGA-AN-A0FL** | **0.09(-)** | **1.07 (-)** | **15.07** | **1+** | **+** |  | **non-TNBC** | **21.24** |
| TCGA-E9-A1ND | 1.44(-) | 0.05 (-) | 13.05 |  | + |  | non-TNBC | 20.85 |
| TCGA-A1-A0SB | 3.16(+) | 0.03 (-) | 32.35 |  | - |  | non-TNBC | 20.71 |
| TCGA-BH-A0DL | 6.99(+) | 0.04 (-) | 9.92 |  | - |  | non-TNBC | 19.54 |
| TCGA-A2-A25F | 0.62(-) | 0.23 (+) | 5.19 |  | - |  | non-TNBC | 18.52 |
| TCGA-A2-A4S1 | 0.29(+) | 0.01 (-) | 0.61 |  | - |  | non-TNBC | 10.53 |
| TCGA-BH-A6R9 | 0.59(-) | 0.25 (+) | 8.18 |  | - |  | non-TNBC | 10.01 |
| **TCGA-JL-A3YW** | **0.35(+)** | **0.09 (+)** | **31.47** | **1+** | **+** |  | **non-TNBC** | **7.87** |
| TCGA-AO-A1KO | 10.78(+) | 9.12 (+) | 14.91 | 1+ | - |  | non-TNBC | 7.51 |
| TCGA-D8-A1JK | 0.40(-) | 0.72 (+) | 22.19 | 1+ | - |  | non-TNBC | 7.43 |
| TCGA-AR-A1AO | 1.47(+) | 1.13 (-) | 14.89 | 1+ | - |  | non-TNBC | 7.20 |
| TCGA-UU-A93S | 0.30(-) | 0.12 (-) | 1668.35 | 3+ | + |  | non-TNBC | 6.87 |
| TCGA-AN-A0AM | 73.18(+) | 0.09 (-) | 12.23 |  | - |  | non-TNBC | 6.80 |
| TCGA-OL-A5RY | 0.99(+) | 0.38(-) | 658.80 |  |  | + | non-TNBC | 6.15 |
| TCGA-E2-A1B0 | 0.14(-) | 0.26(-) | 563.81 | 3+ | + |  | non-TNBC | 6.11 |
| TCGA-D8-A13Y | 15.48(+) | 4.17(+) | 4.83 | 1+ | - |  | non-TNBC | 5.55 |
| TCGA-C8-A12P | 0.15(-) | 0.20(-) | 259.71 | 3+ | + |  | non-TNBC | 5.49 |
| TCGA-LL-A73Z | 7.19(+) | 2.10(+) | 28.34 | 2+ | Equiv | - | non-TNBC | 5.06 |
| TCGA-C8-A3M7 | 4.27(-) | 0.76(-) | 25.47 |  | - |  | TNBC | 5.05 |
| TCGA-GM-A2DI | 23.49(-) | 12.05(-) | 20.30 |  |  | - | TNBC | 5.03 |
| TCGA-C8-A135 | 0.28(-) | 0.19(-) | 433.37 | 3+ | + |  | non-TNBC | 4.99 |
| TCGA-BH-A1FN | 14.34(+) | 3.30(+) | 10.64 |  |  |  | non-TNBC | 4.73 |
| TCGA-A2-A0CY | 52.48(+) | 0.33(+) | 16.84 | 1+ | - | - | non-TNBC | 4.67 |
| TCGA-AC-A2FK | 4.44(+) | 18.20(+) | 58.01 |  |  | - | non-TNBC | 4.53 |
| TCGA-AR-A24U | 1.22(-) | 0.26(-) | 410.17 | 3+ | + |  | non-TNBC | 4.28 |
| TCGA-AN-A04C | 1.97(-) | 0.04(-) | 692.55 | 3+ | + |  | non-TNBC | 4.15 |
| TCGA-BH-A0EE | 1.05(-) | 0.01(-) | 182.51 | 3+ | + |  | non-TNBC | 4.04 |
| TCGA-D8-A1XT | 0.30(-) | 0.13(-) | 692.72 | 3+ | + |  | non-TNBC | 3.98 |
| TCGA-B6-A0RQ | 23.13(+) | 15.83(+) | 45.71 |  |  |  | non-TNBC | 3.87 |
| TCGA-BH-A28O | 5.81(+) | 1.73(+) | 38.57 |  | - |  | non-TNBC | 3.72 |
| TCGA-BH-A1EW | 29.98(-) | 18.90(-) | 42.47 |  | - |  | TNBC | 3.65 |
| TCGA-LD-A9QF | 0.56(-) | 0.20(-) | 812.04 | 3+ | + |  | non-TNBC | 3.46 |
| TCGA-E9-A1N9 | 75.75(-) | 0.50(+) | 20.24 |  | + |  | non-TNBC | 3.27 |
| TCGA-EW-A1PC | 22.20(+) | 0.56(+) | 52.95 | 1+ | - |  | non-TNBC | 3.25 |
| TCGA-E9-A1NA | 37.65(+) | 0.22(+) | 20.39 |  | + |  | non-TNBC | 3.19 |
| TCGA-AC-A3W5 | 1.98(+) | 0.38(+) | 858.10 |  |  | + | non-TNBC | 2.44 |
| TCGA-Z7-A8R5 | 11.43(+) | 1.38(+) | 36.95 |  | - |  | non-TNBC | 2.43 |
| TCGA-E2-A14P | 0.38(-) | 0.12(-) | 877.43 | 3+ | + |  | non-TNBC | 1.99 |
| TCGA-EW-A1PG | 13.18(+) | 7.09(+) | 36.07 | 2+ | Equiv | - | non-TNBC | 1.95 |

*: Perres, abstract value of Pearson residual.

Table S4-2 Forty-three outliers detected using enetLTS for the TNBC dataset in Segaert P, et al. ^[2]^

(all within the 68 outliers detected in table S2-1)

| TCGA-E9-A22G, TCGA-A2-A0YJ, TCGA-A7-A13E, TCGA-A2-A04U, TCGA-AR-A0TP, TCGA-AN-A0FX, TCGA-AR-A251, TCGA-LL-A6FR,TCGA-AC-A62X, TCGA-BH-A5IZ, TCGA-OL-A5S0, TCGA-LL-A5YP, TCGA-LL-A8F5, TCGA-B6-A0IJ, TCGA-A7-A13D, TCGA-AR-A24Q, TCGA-S3-AA0Z, TCGA-AN-A0FJ, TCGA-AR-A1AH, TCGA-D8-A1JM, TCGA-E9-A1NC, TCGA-A2-A3Y0 , TCGA-D8-A1XW, TCGA-AR-A1AJ, TCGA-A2-A1G1, TCGA-AO-A0JL, TCGA-A2-A4RX, TCGA-E2-A14Y, TCGA-E2-A1II, TCGA-AN-A0FL, TCGA-E9-A1ND, TCGA-A1-A0SB, TCGA-BH-A0DL, TCGA-A2-A25F, TCGA-A2-A4S1, TCGA-BH-A6R9, TCGA-JL-A3YW, TCGA-AO-A1KO, TCGA-D8-A1JK, TCGA-AR-A1AO, TCGA-UU-A93S, TCGA-E2-A1B0, TCGA-D8-A13Y |
| --- |

Table S4-5 Sixty-six outliers detected by enetLTS for analysis for dataset with 5000 variables selected randomly(individuals highlighted in bold are suspect individuals) .^*^

| TCGA-A2-A0YJ, TCGA-E9-A22G, TCGA-A7-A13E, TCGA-AR-A0TP, TCGA-LL-A6FR, TCGA-BH-A5IZ, TCGA-OL-A5S0, TCGA-E9-A1NC, **TCGA-AN-A0FX**, TCGA-AC-A62X, TCGA-S3-AA0Z, TCGA-B6-A0IJ, **TCGA-A2-A04U**, **TCGA-AN-A0FJ**, TCGA-AR-A251, **TCGA-LL-A5YP**, TCGA-D8-A1JM, TCGA-D8-A1XW, TCGA-A7-A13D, TCGA-AR-A1AH, TCGA-E2-A14Y, TCGA-LL-A8F5, TCGA-AR-A24Q, **TCGA-AO-A0JL**, TCGA-A2-A3Y0, TCGA-E2-A1II, TCGA-BH-A0DL, TCGA-A2-A4RX, TCGA-AR-A1AJ, TCGA-E9-A1ND, **TCGA-AN-A0FL**, TCGA-A2-A1G1, TCGA-A1-A0SB, TCGA-A2-A25F, TCGA-A2-A4S1, TCGA-BH-A6R9, TCGA-D8-A1JK, TCGA-AO-A1KO, TCGA-BH-A0EE, TCGA-E2-A1B0, TCGA-OL-A5RY, TCGA-C8-A12P, TCGA-A2-A0CY, TCGA-LL-A73Z, TCGA-AR-A1AO, **TCGA-JL-A3YW**, TCGA-D8-A13Y, TCGA-AN-A0AM, TCGA-UU-A93S, TCGA-AN-A04C, TCGA-BH-A1FN, TCGA-BH-A1EW, TCGA-D8-A1XT, TCGA-EW-A1PC, TCGA-A2-A3XW, TCGA-AR-A24U, TCGA-AO-A0JC, TCGA-Z7-A8R5, TCGA-GM-A2DI, TCGA-AC-A2FK, TCGA-B6-A0RQ, TCGA-AO-A12D, TCGA-E9-A1N9, TCGA-C8-A3M7, TCGA-BH-A209, TCGA-E2-A14P |
| --- |

* Of these, 62 are in common with 68 outliers detected in the original TNBC dataset.

T**able** S4- 6 Genes selected by enetLTS for the original TNBC dataset.

| **Gene ID** | **Coefficient** | **Gene ID** | **Coefficient** | **Gene ID** | **Coefficient** | **Gene ID** | **Coefficient** | **Gene ID** | **Coefficient** |
| --- | --- | --- | --- | --- | --- | --- | --- | --- | --- |
| ENSG00000129514 | -0.2763 | ENSG00000196196 | 0.0569 | ENSG00000134873 | 0.03 | ENSG00000167703 | 0.015 | ENSG00000187260 | -0.0035 |
| ENSG00000124664 | -0.2639 | ENSG00000104213 | -0.0552 | ENSG00000035115 | 0.0299 | ENSG00000154719 | 0.015 | ENSG00000259075 | 0.0033 |
| ENSG00000102243 | 0.2447 | ENSG00000078018 | 0.0547 | ENSG00000006652 | 0.0298 | ENSG00000159763 | -0.0149 | ENSG00000139842 | 0.0032 |
| ENSG00000198729 | 0.2322 | ENSG00000026559 | 0.0546 | ENSG00000131386 | -0.0296 | ENSG00000109944 | -0.0147 | ENSG00000103546 | 0.0029 |
| ENSG00000182175 | 0.2129 | ENSG00000162086 | 0.0537 | ENSG00000092068 | -0.0296 | ENSG00000108819 | -0.0141 | ENSG00000161642 | -0.0028 |
| ENSG00000204019 | 0.2075 | ENSG00000163661 | 0.0537 | ENSG00000099282 | -0.0296 | ENSG00000262406 | 0.0141 | ENSG00000214194 | 0.0027 |
| ENSG00000173890 | -0.1966 | ENSG00000137310 | 0.0534 | ENSG00000131089 | 0.0296 | ENSG00000196235 | 0.0139 | ENSG00000172137 | 0.0027 |
| ENSG00000163064 | 0.1837 | ENSG00000135374 | 0.0533 | ENSG00000168542 | -0.0295 | ENSG00000204248 | 0.0138 | ENSG00000138796 | 0.0025 |
| ENSG00000174607 | 0.1821 | ENSG00000198522 | 0.0532 | ENSG00000141858 | 0.0293 | ENSG00000115507 | 0.0137 | ENSG00000198064 | -0.0025 |
| ENSG00000054598 | 0.1781 | ENSG00000134215 | -0.0527 | ENSG00000186185 | 0.0292 | ENSG00000107175 | 0.0137 | ENSG00000244411 | -0.0024 |
| ENSG00000198732 | 0.1728 | ENSG00000108298 | -0.0525 | ENSG00000175895 | -0.0291 | ENSG00000124243 | -0.0137 | ENSG00000104738 | 0.0022 |
| ENSG00000107485 | -0.1714 | ENSG00000183779 | -0.0518 | ENSG00000125820 | -0.0289 | ENSG00000203972 | -0.0136 | ENSG00000213088 | -0.002 |
| ENSG00000143452 | 0.1697 | ENSG00000168899 | -0.0515 | ENSG00000143797 | 0.0288 | ENSG00000151379 | -0.0135 | ENSG00000256162 | 0.002 |
| ENSG00000213064 | 0.1612 | ENSG00000131748 | -0.0515 | ENSG00000184599 | 0.0283 | ENSG00000128918 | -0.0134 | ENSG00000140832 | 0.0018 |
| ENSG00000074410 | -0.1552 | ENSG00000186897 | 0.0514 | ENSG00000103260 | -0.0281 | ENSG00000100522 | -0.0134 | ENSG00000184911 | -0.0018 |
| ENSG00000160392 | 0.1467 | ENSG00000006555 | 0.0509 | ENSG00000175899 | -0.0281 | ENSG00000137501 | -0.0133 | ENSG00000088325 | 0.0017 |
| ENSG00000162949 | -0.1426 | ENSG00000186204 | -0.0505 | ENSG00000165124 | -0.028 | ENSG00000136114 | -0.0132 | ENSG00000225556 | 0.0017 |
| ENSG00000163879 | -0.1419 | ENSG00000132823 | 0.0498 | ENSG00000215218 | -0.028 | ENSG00000142192 | 0.0131 | ENSG00000167992 | -0.0017 |
| ENSG00000102854 | 0.1377 | ENSG00000117153 | -0.0491 | ENSG00000107796 | -0.0277 | ENSG00000086289 | -0.0129 | ENSG00000164574 | -0.0016 |
| ENSG00000138449 | -0.1353 | ENSG00000085465 | -0.0485 | ENSG00000138829 | 0.0272 | ENSG00000166535 | 0.0127 | ENSG00000141569 | 0.0013 |
| ENSG00000112812 | 0.1292 | ENSG00000184675 | 0.0483 | ENSG00000142530 | 0.0272 | ENSG00000182742 | -0.0126 | ENSG00000184454 | 0.0011 |
| ENSG00000106541 | -0.1286 | ENSG00000117148 | 0.048 | ENSG00000146233 | 0.027 | ENSG00000121775 | 0.0124 | ENSG00000110427 | 0.0011 |
| ENSG00000160180 | -0.1208 | ENSG00000178363 | -0.0477 | ENSG00000144228 | -0.0259 | ENSG00000107798 | -0.012 | ENSG00000188536 | -0.001 |
| ENSG00000167771 | 0.1202 | ENSG00000111328 | 0.0472 | ENSG00000186868 | -0.0258 | ENSG00000152977 | 0.0119 | ENSG00000176658 | -0.0009 |
| ENSG00000102302 | 0.1198 | ENSG00000095752 | -0.0469 | ENSG00000176890 | 0.0257 | ENSG00000160691 | 0.0119 | ENSG00000080546 | -0.0009 |
| ENSG00000261857 | 0.1187 | ENSG00000065371 | 0.0469 | ENSG00000139211 | -0.0255 | ENSG00000198952 | 0.0118 | ENSG00000162493 | -0.0008 |
| ENSG00000172602 | -0.1176 | ENSG00000134755 | 0.0468 | ENSG00000185352 | -0.0255 | ENSG00000125810 | -0.0117 | ENSG00000075643 | 0.0006 |
| ENSG00000148468 | 0.1163 | ENSG00000164975 | 0.0461 | ENSG00000111057 | -0.0255 | ENSG00000049130 | -0.0117 | ENSG00000169436 | 0.0006 |
| ENSG00000186832 | 0.1148 | ENSG00000197253 | -0.046 | ENSG00000040608 | 0.0254 | ENSG00000161040 | 0.0116 | ENSG00000119787 | 0.0005 |
| ENSG00000108244 | 0.1115 | ENSG00000167614 | 0.0455 | ENSG00000107562 | -0.0254 | ENSG00000118655 | 0.0113 | ENSG00000162078 | -0.0005 |
| ENSG00000161395 | -0.11 | ENSG00000115648 | -0.0454 | ENSG00000109436 | -0.025 | ENSG00000175183 | 0.0112 | ENSG00000184661 | 0.0005 |
| ENSG00000173267 | -0.1099 | ENSG00000161542 | 0.0452 | ENSG00000066279 | 0.0249 | ENSG00000132386 | -0.0112 | ENSG00000128709 | -0.0005 |
| ENSG00000164626 | 0.1085 | ENSG00000166682 | 0.0452 | ENSG00000142507 | 0.0248 | ENSG00000163472 | 0.0112 | ENSG00000124107 | 0.0001 |
| ENSG00000152137 | -0.1084 | ENSG00000169131 | 0.0451 | ENSG00000136928 | 0.0248 | ENSG00000167644 | -0.0111 |  |  |
| ENSG00000204385 | -0.0996 | ENSG00000127337 | -0.0451 | ENSG00000196208 | -0.0247 | ENSG00000171798 | -0.0111 |  |  |
| ENSG00000072858 | -0.0973 | ENSG00000168386 | -0.045 | ENSG00000104332 | 0.0247 | ENSG00000204310 | 0.0111 |  |  |
| ENSG00000092758 | 0.0971 | ENSG00000185634 | 0.0446 | ENSG00000106266 | 0.0246 | ENSG00000162437 | 0.011 |  |  |
| ENSG00000158164 | 0.0964 | ENSG00000166439 | 0.0445 | ENSG00000115255 | -0.0244 | ENSG00000006282 | -0.0109 |  |  |
| ENSG00000123342 | -0.0939 | ENSG00000198848 | -0.0437 | ENSG00000170920 | 0.0244 | ENSG00000120063 | -0.0109 |  |  |
| ENSG00000173467 | -0.0934 | ENSG00000167861 | -0.0435 | ENSG00000122705 | 0.0243 | ENSG00000115825 | 0.0108 |  |  |
| ENSG00000169083 | -0.0933 | ENSG00000064225 | 0.0434 | ENSG00000111231 | -0.0243 | ENSG00000014641 | 0.0107 |  |  |
| ENSG00000188763 | 0.0933 | ENSG00000183401 | -0.0433 | ENSG00000117394 | 0.0242 | ENSG00000162627 | 0.0104 |  |  |
| ENSG00000152455 | 0.0921 | ENSG00000074211 | -0.0433 | ENSG00000154096 | -0.0242 | ENSG00000197457 | -0.0104 |  |  |
| ENSG00000163683 | -0.092 | ENSG00000021300 | 0.0433 | ENSG00000111319 | -0.0237 | ENSG00000154640 | 0.0101 |  |  |
| ENSG00000143401 | 0.0919 | ENSG00000102554 | 0.043 | ENSG00000128595 | 0.0231 | ENSG00000280447 | 0.0101 |  |  |
| ENSG00000163975 | 0.091 | ENSG00000042493 | 0.043 | ENSG00000152642 | -0.023 | ENSG00000171431 | -0.01 |  |  |
| ENSG00000171862 | -0.0907 | ENSG00000181481 | -0.0429 | ENSG00000126653 | -0.023 | ENSG00000116212 | 0.0099 |  |  |
| ENSG00000132561 | -0.0895 | ENSG00000151617 | -0.0428 | ENSG00000100100 | -0.0229 | ENSG00000163412 | -0.0095 |  |  |
| ENSG00000133392 | -0.0889 | ENSG00000173926 | 0.0417 | ENSG00000183765 | 0.0228 | ENSG00000171227 | -0.009 |  |  |
| ENSG00000164106 | 0.0872 | ENSG00000176597 | 0.0416 | ENSG00000004799 | -0.0227 | ENSG00000167779 | -0.009 |  |  |
| ENSG00000155918 | 0.0868 | ENSG00000056277 | 0.0411 | ENSG00000134830 | -0.0227 | ENSG00000059378 | 0.0088 |  |  |
| ENSG00000155622 | 0.0854 | ENSG00000072041 | 0.0409 | ENSG00000110484 | -0.0225 | ENSG00000163293 | 0.0084 |  |  |
| ENSG00000100167 | 0.0853 | ENSG00000189129 | -0.0406 | ENSG00000188959 | -0.0223 | ENSG00000110090 | -0.0083 |  |  |
| ENSG00000185915 | 0.0844 | ENSG00000125398 | 0.0406 | ENSG00000100473 | 0.0223 | ENSG00000204574 | 0.0081 |  |  |
| ENSG00000146013 | 0.0844 | ENSG00000078403 | 0.0404 | ENSG00000102317 | -0.0219 | ENSG00000019169 | 0.008 |  |  |
| ENSG00000138801 | 0.0833 | ENSG00000167680 | -0.0404 | ENSG00000137547 | 0.0218 | ENSG00000198523 | -0.0079 |  |  |
| ENSG00000064886 | 0.0821 | ENSG00000013619 | 0.0404 | ENSG00000106780 | -0.0216 | ENSG00000165912 | 0.0079 |  |  |
| ENSG00000204175 | 0.079 | ENSG00000147526 | -0.0403 | ENSG00000125355 | -0.0212 | ENSG00000101955 | -0.0077 |  |  |
| ENSG00000164434 | 0.0782 | ENSG00000160182 | -0.0402 | ENSG00000166851 | 0.0212 | ENSG00000154429 | 0.0076 |  |  |
| ENSG00000159352 | 0.0782 | ENSG00000107281 | -0.0401 | ENSG00000267748 | 0.0208 | ENSG00000026297 | 0.0075 |  |  |
| ENSG00000170579 | 0.0767 | ENSG00000117152 | -0.0401 | ENSG00000108798 | -0.0208 | ENSG00000106392 | 0.0075 |  |  |
| ENSG00000181019 | -0.0765 | ENSG00000138639 | -0.0399 | ENSG00000124608 | 0.0207 | ENSG00000088926 | 0.0075 |  |  |
| ENSG00000176532 | -0.0763 | ENSG00000256229 | -0.0397 | ENSG00000198535 | -0.0204 | ENSG00000109472 | -0.0074 |  |  |
| ENSG00000140511 | 0.0757 | ENSG00000003989 | -0.0395 | ENSG00000124702 | 0.0203 | ENSG00000046604 | 0.0074 |  |  |
| ENSG00000078246 | 0.0756 | ENSG00000131149 | -0.0394 | ENSG00000273291 | 0.0203 | ENSG00000162894 | -0.0072 |  |  |
| ENSG00000072954 | 0.0749 | ENSG00000080608 | 0.0389 | ENSG00000159784 | -0.0201 | ENSG00000137309 | 0.0068 |  |  |
| ENSG00000121577 | -0.0731 | ENSG00000153404 | 0.0386 | ENSG00000183807 | -0.02 | ENSG00000170425 | 0.0067 |  |  |
| ENSG00000165186 | 0.0728 | ENSG00000117399 | 0.0385 | ENSG00000163697 | -0.02 | ENSG00000177359 | 0.0066 |  |  |
| ENSG00000151461 | 0.0726 | ENSG00000085999 | 0.0382 | ENSG00000145555 | 0.0194 | ENSG00000173621 | 0.0066 |  |  |
| ENSG00000169908 | 0.0706 | ENSG00000168350 | -0.0381 | ENSG00000204262 | -0.0193 | ENSG00000093009 | 0.0065 |  |  |
| ENSG00000151117 | -0.0696 | ENSG00000204371 | 0.0379 | ENSG00000137504 | -0.0192 | ENSG00000151365 | -0.0064 |  |  |
| ENSG00000114547 | 0.069 | ENSG00000081923 | -0.0379 | ENSG00000160213 | 0.0192 | ENSG00000240857 | 0.0064 |  |  |
| ENSG00000166575 | -0.069 | ENSG00000141639 | 0.0377 | ENSG00000204348 | 0.0191 | ENSG00000172057 | -0.0064 |  |  |
| ENSG00000101311 | 0.0686 | ENSG00000160678 | 0.0376 | ENSG00000008283 | -0.019 | ENSG00000161921 | 0.0061 |  |  |
| ENSG00000107159 | 0.0675 | ENSG00000163431 | -0.0375 | ENSG00000170734 | 0.0188 | ENSG00000135637 | 0.006 |  |  |
| ENSG00000158315 | 0.0671 | ENSG00000150687 | -0.0375 | ENSG00000136944 | -0.0183 | ENSG00000138764 | -0.0059 |  |  |
| ENSG00000156219 | 0.0657 | ENSG00000130176 | -0.0373 | ENSG00000110583 | 0.0181 | ENSG00000187045 | -0.0059 |  |  |
| ENSG00000177614 | 0.0652 | ENSG00000107807 | 0.0371 | ENSG00000174640 | -0.0179 | ENSG00000083444 | 0.0059 |  |  |
| ENSG00000196646 | -0.0644 | ENSG00000116299 | -0.0367 | ENSG00000126453 | 0.0178 | ENSG00000107099 | -0.0059 |  |  |
| ENSG00000115241 | 0.0643 | ENSG00000169047 | -0.0363 | ENSG00000100448 | -0.0178 | ENSG00000184898 | -0.0058 |  |  |
| ENSG00000091831 | -0.0641 | ENSG00000100078 | -0.0358 | ENSG00000166086 | -0.0176 | ENSG00000177839 | 0.0055 |  |  |
| ENSG00000151715 | -0.0635 | ENSG00000111846 | 0.0357 | ENSG00000105855 | 0.0175 | ENSG00000138615 | -0.0055 |  |  |
| ENSG00000143878 | -0.0634 | ENSG00000164695 | 0.0352 | ENSG00000213085 | -0.0175 | ENSG00000149573 | 0.0054 |  |  |
| ENSG00000114745 | 0.0631 | ENSG00000196917 | -0.0351 | ENSG00000112902 | -0.0173 | ENSG00000175764 | -0.0049 |  |  |
| ENSG00000176055 | -0.0629 | ENSG00000127920 | -0.0347 | ENSG00000182263 | 0.0173 | ENSG00000102384 | 0.0048 |  |  |
| ENSG00000121957 | 0.0625 | ENSG00000066322 | 0.0345 | ENSG00000171954 | -0.0172 | ENSG00000089327 | 0.0046 |  |  |
| ENSG00000094755 | 0.0623 | ENSG00000139083 | 0.0343 | ENSG00000140682 | -0.017 | ENSG00000082175 | -0.0046 |  |  |
| ENSG00000204542 | 0.0621 | ENSG00000167733 | 0.0342 | ENSG00000073792 | 0.0167 | ENSG00000135842 | 0.0046 |  |  |
| ENSG00000125378 | -0.0616 | ENSG00000135069 | 0.0342 | ENSG00000139055 | 0.0166 | ENSG00000115009 | 0.0046 |  |  |
| ENSG00000253958 | 0.0616 | ENSG00000050628 | -0.0342 | ENSG00000070018 | 0.0165 | ENSG00000177363 | -0.0046 |  |  |
| ENSG00000150630 | -0.0608 | ENSG00000008196 | -0.034 | ENSG00000144647 | 0.0163 | ENSG00000167323 | 0.0043 |  |  |
| ENSG00000138101 | 0.0606 | ENSG00000173917 | -0.0336 | ENSG00000197763 | 0.0163 | ENSG00000124587 | 0.0039 |  |  |
| ENSG00000125691 | -0.0605 | ENSG00000172551 | -0.0336 | ENSG00000179165 | 0.0162 | ENSG00000128294 | -0.0039 |  |  |
| ENSG00000274997 | 0.0599 | ENSG00000132164 | 0.0327 | ENSG00000145604 | 0.0158 | ENSG00000101443 | 0.0039 |  |  |
| ENSG00000127418 | 0.0597 | ENSG00000142973 | -0.0318 | ENSG00000138080 | -0.0157 | ENSG00000141744 | -0.0038 |  |  |
| ENSG00000152284 | 0.0593 | ENSG00000179715 | -0.0317 | ENSG00000148339 | -0.0157 | ENSG00000085491 | 0.0037 |  |  |
| ENSG00000187741 | 0.0585 | ENSG00000153551 | 0.0314 | ENSG00000137077 | -0.0156 | ENSG00000065618 | -0.0037 |  |  |
| ENSG00000088899 | 0.0581 | ENSG00000167065 | -0.0307 | ENSG00000132932 | -0.0154 | ENSG00000163485 | 0.0036 |  |  |
| ENSG00000141741 | -0.0581 | ENSG00000172236 | -0.0302 | ENSG00000167755 | 0.0153 | ENSG00000276293 | -0.0035 |  |  |
| ENSG00000147536 | 0.0572 | ENSG00000105699 | 0.0301 | ENSG00000176490 | 0.0153 | ENSG00000120262 | -0.0035 |  |  |

# S5. Results of Rlogreg for the Analysis of TNBC dataset

**Table S**5-1 One hundred and nine outliers detected using Rlogregfor the TNBC dataset (individuals highlighted in bold are suspect individuals.)

| **TCGA-AC-A8OS**, TCGA-BH-A201, TCGA-AR-A1AO, TCGA-BH-A6R9, TCGA-AC-A62X, TCGA-A2-A0YJ, TCGA-AO-A0JC, TCGA-A2-A25A, **TCGA-LL-A5YP**, TCGA-AC-A3TM, TCGA-A7-A13D, TCGA-E2-A1II, TCGA-AR-A1AH, TCGA-AC-A2FK, TCGA-D8-A4Z1, TCGA-AC-A2FF, TCGA-D8-A1XT, TCGA-A2-A4S3, TCGA-A7-A426, TCGA-A2-A3XW, TCGA-E2-A14Y, TCGA-BH-A202, TCGA-A8-A07U, TCGA-BH-A8G0, TCGA-LD-A74U, TCGA-GM-A4E0, **TCGA-AO-A0JL**, **TCGA-AN-A0FL**, TCGA-E9-A6HE, TCGA-AO-A1KO, TCGA-A2-A0EP, **TCGA-AN-A0FX**, TCGA-A8-A08X, TCGA-A1-A0SB, TCGA-A8-A08L, TCGA-BH-A0BO, TCGA-EW-A1J2, TCGA-A2-A04X, TCGA-A2-A0EW, TCGA-LL-A73Z, TCGA-AO-A12D, TCGA-A2-A0CO, TCGA-E2-A3DX, TCGA-A2-A3KC, TCGA-D8-A1JM, TCGA-E9-A1NC, TCGA-AO-A0J9, TCGA-XX-A89A, TCGA-EW-A1J6, TCGA-A2-A1G1, TCGA-BH-A28O, TCGA-LL-A6FR, TCGA-A2-A3Y0, TCGA-A8-A08H, TCGA-BH-A0HF, TCGA-E2-A108, TCGA-D8-A1Y0, TCGA-A8-A07L, TCGA-B6-A0IJ, TCGA-AR-A2LQ, TCGA-AO-A0JE, TCGA-E2-A1LB, TCGA-A2-A04W, TCGA-AR-A0TP, TCGA-S3-AA0Z, TCGA-A2-A4S1, TCGA-A2-A0CR, TCGA-A7-A13E, **TCGA-AC-A3YI**, TCGA-C8-A1HF, **TCGA-JL-A3YW**, TCGA-LL-A440, TCGA-E9-A1N9, TCGA-A2-A0YK, TCGA-BH-A0DX, TCGA-E2-A1B5, TCGA-AN-A04C, TCGA-E9-A1ND, TCGA-AC-A3OD, TCGA-AR-A24H, TCGA-B6-A0RQ, TCGA-BH-A1FN, TCGA-3C-AALI, **TCGA-AN-A0FJ**, TCGA-A2-A4RW, TCGA-D8-A1XW, TCGA-BH-A0EE, TCGA-B6-A0IH, TCGA-OL-A5S0, TCGA-E9-A22G, TCGA-AR-A24U, TCGA-A8-A09W, TCGA-AR-A24Q, TCGA-C8-A278, TCGA-E2-A1B0, TCGA-A8-A07R, TCGA-A2-A0EY, TCGA-AR-A251, TCGA-BH-A8FZ, TCGA-A2-A4RX, TCGA-AR-A1AJ, **TCGA-A2-A04U**, TCGA-D8-A27N, TCGA-BH-A5IZ, TCGA-D8-A13Y, TCGA-LL-A8F5, TCGA-BH-A0B7, TCGA-A2-A3XV, TCGA-AN-A0AM |
| --- |

**Reference**

[1] Lopes MB, Verissimo A, Carrasquinha E, Casimiro S, Beerenwinkel N, Vinga S. Ensemble outlier detection and gene selection in triple-negative breast cancer data[J]. BMC Bioinformatics, 2018, 19(1): 168.

[2] Segaert P, Lopes MB, Casimiro S, Vinga S, Rousseeuw PJ. Robust identification of target genes and outliers in triple-negative breast cancer data[J]. Stat Methods Med Res, 2019, 28(10-11): 3042-3056.
